# Enhanced pharmacokinetic and therapeutic of GSH-responsive mPEG-*b*-P(HPMA)-SGI1776 conjugate for osteosarcoma

**DOI:** 10.3389/fphar.2025.1676770

**Published:** 2025-10-23

**Authors:** Xingchen Shi, Rourou Wang, Zhenghua Zhang, Zixuan Wang, Weibing Xu, Xinhe Shi, Zhe Geng

**Affiliations:** ^1^ Orthopedics Department of Lanzhou Second People’s Hospital, Lanzhou, China; ^2^ College of Science, Gansu Agricultural University, Lanzhou, China; ^3^ College of Environment and Chemical Engineering, Lanzhou Resources and Environment Voc-Tech University, Lanzhou, China; ^4^ Cuiying Biomedical Research Center of Lanzhou University Second Hospital, Lanzhou, China; ^5^ Medicine School of Lanzhou University, Lanzhou, China

**Keywords:** SGI-1776, GSH-responsive, polymeric prodrug, osteosarcoma, pharmacokinetics

## Abstract

**Introduction:**

SGI-1776 represents the first serine/threonine kinase inhibitor to be utilized in the treatment of osteosarcoma. It is severely restricted by poor aqueous solubility, short degradation half-life and severe side effect.

**Methods:**

A polymeric prodrug is prepared by covalently embedding SGI-1776 into mPEG-*b*-P(N-(2-hydroxypropyl) methylacrylamide) carrier via GSH-responsive disulfide bond. The chemical composition and assembly properties of the conjugate are characterized.

**Results:**

The loading capacity of SGI1776 in the conjugate is 22%. The conjugate displays a typical spherical nanoparticle with diameters around 150–260 nm. Cumulative release amounts of SGI1776 can be detected to be 52.9% at the concentration dithiothreitol of 20 mM at 24 h. The hemolysis rate is about 2.35% even when the concentration increases up to 1,000 mg/L. The value of IC50 is about 18.8 μg SGI1776 equiv. per mL for 143b cells.

**Discussion:**

The conjugate is more likely to induce apoptosis and can be blocked 143b cells in the S cycle phase. The maximum plasma concentration of the conjugate is attained 0.52 ± 0.092 μg/mL at 4 ± 0 h after the oral administration. The conjugate exhibits better lysosomal escape ability, biocompatibility.

## 1 Introduction

Osteosarcoma is a malignant bone tumor often accompanied by tumor-like bone matrix formation, with a peak incidence in adolescence (Valery et al., 2014). Due to its high systemic spread rate, lung metastasis and distal bone metastasis occur in most patients. At present, the clinically standardized treatment of osteosarcoma includes surgery and combined neoadjuvant chemotherapy (Kim et al., 2024). However, due to the complex anatomical structure of bone and the lack of clear boundaries between invasive tumors and surrounding tissues, it is difficult to remove bone tumors without damaging the bone matrix during surgery and is often accompanied by a high risk of recurrence and metastasis, requiring functional reconstruction in patients with metastasis (Fox and Trotta, 2013; Schmolders et al., 2017). The systemic toxicity of chemotherapy drugs prompts researchers to explore new strategies to address this intractable problem (Yang et al., 2018).

One of the newer drug categories that have been utilized to address the problems mentioned before are SGI1776. Provirus integration site for Moloney murine leukemia virus (PIM) kinases are a family of serine/threonine kinases (Aziz et al., 2020). The PIM kinase family plays numerous roles in apoptosis, cell cycle progression, motility, and chemoresistance in pediatric solid tumors (Nawijn et al., 2011). Therefore, PIM kinases inhibitors have been identified as potential therapeutic targets in treatment of osteosarcoma. SGI-1776 is the first PIM kinase inhibitor employed in a human clinical trial. SGI-1776 mainly inhibits the catalytic activity of PIM kinase by blocking its binding to ATP (Xu et al., 2014). However, the clinical trial and application of SGI-1776 is severely restricted by poor aqueous solubility, short degradation half-life and severe side effect (Serrano-Saenz et al., 2019; Xu et al., 2022).

In recent years, responsive nanodrug delivery systems in tumor microenvironments such as low pH and high reactive oxygen species (ROS) level have been developed for delivery anticancer agents to tumor site via enhanced permeability and retention (EPR) effect (Chen et al., 2022; Liu et al., 2025; Zhang et al., 2022; Zhang et al., 2024), which provides important ways to overcome the shortcomings of these small molecule inhibitors. pH-responsive calcium carbonate -crosslinked hyaluronate nanoparticles/CDDP nanogel to deliver DOX for the treatment of osteosarcoma is reported. The two nanoplatforms exhibit acid-sensitive drug release, enhanced antitumor efficacy, and reduced adverse effects (Liao et al., 2024; Liu et al., 2017; Zhang Y. et al., 2018). Novel ROS-responsive polymeric drug nanoparticles with a PEG shell are developed to prolong blood circulation time and promoting tumor-targeting efficiency and tissue penetration, which inhibited tumor growth *in vitro* and *in vivo* in osteosarcoma treatment (Xu et al., 2017). On the other hand, the GSH level within tumor cells is as high as 2–20 mM, which is 100–1000 times and 7–10 times that of extracellular matrix and normal tissue, respectively (Aluri et al., 2009; Yao et al., 2019). Xu et al. ligated doxorubicin (DOX) to polyethylene glycol (PEG) through cis-aconitic anhydride (CA) and disulfide bonds (SS) to obtain the PEG-SS-CA-DOX prodrug (Xu J. et al., 2023). PEG-SS-CA-DOX micelles achieved efficient and rapid release of DOX under the dual stimulation of weak acidic pH and high GSH content of tumors, with a high inhibition rate of 70% against breast cancer cells. Xu et al. prepared nanoparticles (FA-CMC-GNA NPs) using an emulsion solvent evaporation method, with a disulfide bond-crosslinked thiolated carboxymethyl cellulose as the backbone, encapsulating the hydrophobic drug gambogenic acid (Xu et al., 2025). The FA-CMC-GNA NPs released gambogenic acid in response to GSH, which resulted in a significant inhibitory effect on A549 cells. Therefore, the GSH can trigger release of therapeutic molecules in osteosarcoma cell, especially for the prodrug compounds which contain disulfide bond (Liu et al., 2021). A large number of GSH-responsive drug delivery systems have been fabricated to improve the efficiency of drug utilization (Hsu and Almutairi, 2021; Pillarisetti et al., 2023). Though a small molecule inhibitor of PIM1, QCAi, was conjugated to Cy7 to enhance its solubility and simultaneously endow it with imaging properties (Yi et al., 2021), studies on GSH-responsive SGI-1776 delivery and controlled release nanomedicine systems for the treatment of osteosarcoma have not been reported yet.

Our research group has long studied the use of Poly (N-(2-hydroxypropyl) methacrylamide) (PHPMA) as a drug carrier to deliver various small molecule drugs (Xu et al., 2019). P(HPMA) is a water-soluble, non-charged, nontoxic, non-immunogenic, and biocompatible polymer (Li et al., 2021). Compared with PEG and PLGA which have fewer modifiable groups, P(HPMA) contains a large amount of hydroxyl moieties that can be utilized as handles for installation of various drugs via ester linkages, affording potentially-responsive delivery systems (Alfurhood et al., 2016). Compared with positively charged PEI and PMAMA, P(HPMA) exhibits negative charge and non-immunogenicity, demonstrating good biocompatibility (Krakovičová et al., 2009). In addition, the conjugation strategy improves drug utilization and bioavailability by enhancing solubility, prolonging circulation, and reducing systemic toxicity. Herein, the PIM inhibitor SGI1776 is linked to mPEG-*b*-P(HPMA) via disulfide bond, and the self-assembly and sustained-release properties of the polymer prodrug, as well as the activity of inhibiting 143b cell proliferation *in vitro* are investigated.

## 2 Experimental

### 2.1 Materials and instruments

SGI1776 purchased from Shanghai Hanxiang Biotechnology Co., LTD. mPEG (Mw = 2000), dicyclohexyl carbodiimide (DCC), 4-dimethylaminopyridine (DMAP), β-hydroxypropyl methacrylate (HPMA), azodiisobutyronitrile, cck-8 assay kit, fluorescent dye Cy5.5, 4-cyano-4-(dodecylsulfanylthiocarbonyl) sulfanyl pentanoic acid, dithiothreitol (DTT) and 4, 4′-dithiodibutyl acid purchased from Shanghai Macklin Biochemical Technology Co., LTD. NMR spectra were recorded on Agilent Technologies 600 (Agilent DD2-600 MHz) instruments. The infrared spectra were performed on a Digilab FTS-3000 FTIR spectrophotometer. Photoluminescence spectra were performed on a Shimadzu TU-1901 dual beam UV-Vis spectrophotometer. The morphologies were characterized using an S-4800 field emission scanning electron microscopy (SEM) (HITACHI, Tokyo, Japan).

### 2.2 Synthesis of mPEG-*b*-P(HPMA)

A solution of 4-cyano-4-(dodecylsulfanylthiocarbonyl)sulfanyl pentanoic acid (0.08 g, 0.3 mmol) in anhydrous DCM (20 mL) was introduced in a dry flask containing mPEG113 (1.0 g, 1 mmol). Then a solution of DCC (0.6 g, 0.3 mmol) and DMAP (0.004 g, 0.01 mmol) in anhydrous DCM (10 mL) was added dropwise to the reaction mixture at 0 °C. The esterification reaction proceeded with stirring at room temperature for 48 h. A large amount of cold ether was added to the reaction solution to collect precipitation. The precipitation was further dissolved in 10 mL of dichloromethane, then dropped with cold ether, and the precipitation was collected again. The collected precipitation was dried in a vacuum oven at 40 °C to obtain a powder (mPEG-CTA). mPEG-b-P(HPMA) was synthesized according to the following experimental steps. mPEG-CTA (0.425 g) and HPMA (0.5 g) were dissolved in 5 mL DMSO. The initiator AIBN (0.022 g) was added. After 30 min of degassing with nitrogen bubble, the solution was polymerized under nitrogen protection at 65 °C for 24 h. The product was precipitated with a mixture of acetone and ether. mPEG-*b*-p (HPMA) was obtained 0.78 g with a yield of 80%.

### 2.3 Synthesis and characterization of mPEG-*b*-P(HPMA)-SS-COOH

mPEG-*b*-P(HPMA) (0.5 g, 0.25 mmol) and 4, 4′-dithiodibutyl acid (0.05 g, 0.2 mmol) were dissolved in 5 mL of DMSO. DCC (0.64 g, 3.1 mmol) and DMAP (20 mg) were added to this solution. The mixture was continuously stirred for 72 h 20 mL of distilled water was added to the reaction mixture, followed by dialysis (MWCO 1500) for 3 days. Change the deionized water outside the dialysis bag every 2 hours. The solution in the dialysis bag is freeze-dried at −60 °C for 48 h to obtain the product denoted as mPEG-*b*-P(HPMA)-SS-COOH, yield: 75.0%.

### 2.4 Synthesis of mPEG-*b*-P(HPMA)-SGI1776 conjugate

mPEG-*b*-P(HPMA)-SS-COOH (0.25 g) and SGI1776 (0.015 g) were dissolved in 5 mL DMSO. DCC (0.023 g) and NHS (0.012 g) was added to the above solution. The reaction mixture was stirred at 25 °C for 24 h precipitation will appear and be filtered after 20 mL of acetone and ether mixture (v/v = 1:1) is added to the reaction mixture. The precipitate was dissolved with anhydrous methanol, centrifuged by an ultrafiltration concentrator with a molecular weight of 3000 to obtain conjugate. 20 mg of the conjugate was dissolved in 0.2 mL of DMSO, and then the solution was slowly added to 5.0 mL of deionized water under stir. After continuous stirring for 5 h, the solution was placed in the dialysis bag of MWCO3500 for dialysis for 12 h, and the water was changed every 2 h to obtain the nanoparticles. The critical association concentration of the conjugate was 1.4 × 10^−4^ g/L.

### 2.5 Redox-responsive release

The performance of SGI1776 release from conjugate was studied via a dialysis method (MWCO 1000) in mediums with DTT concentration of 0.0 and 20 mM at 37 °C. 5 mL conjugate solution (5 mg) was shifted to a dialysis tube and kept in 25 mL PBS solution with Tween-80 (0.5%, w/v). The release medium (1 mL) was collected, and the equivalent amount of external buffer was added to maintain the sink condition at certain intervals. The quantity of released was determined via UV-Vis spectrum. Accordingly, the cumulative release was calculated using the following formula:

$$Q\left(\%\right)=\left(V_{0}C_{t}+V\times \sum_{n=1}^{t-1}C_{t-1}\right)\times 100\%\times W^{-1}\times X^{-1}$$

Ct is the concentration of SGI-1776 in the release medium measured at each time point (mg·mL^-1^); W is the total weight of the conjugate (mg); V0 is the total volume of the release medium; V is the volume of each sample; X is the content of SGI in the conjugate (%). The release of SGI1776 was verified by HPLC. 10 mg of the sample is dissolved in 10 mL of deionized water and evenly divided into two parts. The first sample is not added with DTT as the control, and the other sample was added with DTT (20 mM) as the release medium. 0.5 mL of the release solution was taken at 0, 2, 4, 12, 24, and 48 h respectively, and the release amount of SGI1776 was detected by HPLC. Conditions for HPLC are described in 2.14.

### 2.6 Hemolysis assay

Animal experimental procedures are in accordance with the provisions of the “Experimental Animal Care and Use Guide”, and have been reviewed and approved by the Animal Ethics Committee of Gansu Agricultural University. Typically, 5.0 mL of fresh rabbit blood was centrifuged and washed in a centrifuge tube containing saline repeatedly and until the supernatant became nearly colorless. The obtained red blood cells were prepared into a 2% (v/v) suspension using physiological saline. Hemolysis tests were carried out following established protocols (Ishak et al., 2017). A series of conjugate solutions with varying concentrations and equal volume of 2% (v/v) cell suspension were mixed and placed in tubes. Moreover, distilled water and physiological saline was mixed with cell suspension, serving as positive and negative controls, respectively. All the mixed solutions were centrifuged at 2,000 rpm for 15 min after incubated at 37 °C for 1.0 h. The absorbance value (OD) of the supernatant (100 uL) was measured at a wavelength of 540 nm. The hemolysis rate is obtained using the following formula:

$$Hemolysis\;rate\;\%=\frac{{OD}_{sample\;}-{OD}_{negative\;}}{{OD}_{positive}-{OD}_{negative\;}}\times 100\%$$

### 2.7 *In vitro* activity test

143b cells were inoculated on 96-well plates with a density of 5 × 10^4^/mL and cultured in a 5% CO_2_ incubator at 37 °C for 24 h. The conjugate nanoparticles were added to the 96-well plates in a concentration gradient of 0.05, 0.15, 0.30, 0.60, and 1.2 mg/L. Cells treated with PBS were used as a control group and incubated under same environment. The cell media in the 96-well plates were discarded after adding thiazole blue (5 mg mL^-1^, 20 μL/well) for 4 h, then, DMSO (150 μL) was added to each well and the plate were shaken for 10 min. The absorbance (OD) was recorded at 490 nm under the enzyme standard and cell viability was calculated.

### 2.8 Cell imaging assay

143b cells were inoculated onto six-well plates (1 × 10^5^ cells/well), and then incubated in an incubator at 5% CO_2_ and 37 °C for 24 h. Three separate polymeric nanoparticle solutions were introduced to each well, ensuring that the final concentration of the drugs reached 30 μg/ml. A solution without the polymeric nanoparticles served as the control group. After incubating in 5% CO_2_ at 37 °C for another 24 h, the medium was discarded, and each well was washed twice with PBS. Subsequently, the cells in each well were fixed with 500 μL of fixative for 15 min. After discarding the fixative, staining solution containing propidium iodide (PI) and bisBenzimide H33342 (Hochest33342) was added to in a centrifuge tube, which was then shaken gently for 15 min. After that, the staining solution was discarded. Each well was then washed with PBS three times with 7–8 min for each wash. Finally, 1 mL of PBS was added to each well, and then the cells were examined under a fluorescent microscope.

### 2.9 Endosomal escape

Cells were cultured with Cy5.5-labelled conjugate and the final concentration was selected to be 4 and 10 μg/mL (equivalent SGI1776 dose). The culture medium was removed after 24 h incubation. The lysosome was stained with 100 nmol/L Lyso-Tracker GreenTM for 2.0 h under 35 °C. The cells were washed with PBS for 3 times (1 mL each time), and then fixed with 4% formaldehyde solution for 15 min. The fluorescence images were collected on laser confocal microscope. The excitation wavelengths of Cy5.5-labelled conjugate and Lyso-Tracker GreenTM are 650 and 511 nm, respectively. The degree of overlap between the two types of fluorescence was evaluated by calculating fluorescence colocalization coefficient in ImageJ software.

### 2.10 Cell apoptosis and assay

The annexin V-FITC apoptosis assay kit was employed to quantitatively detect apoptotic and necrotic cells to evaluate the cell death mechanism. The cell culture procedure parallels that of the MTT assay. Cells were collected into centrifuge tubes, after which 300 μL of binding solution was introduced to each tube. Then, 5 μL of Annexin-V was added to each tube. After shielding from light for 15 min, 10 μL of PI was added, and the tubes were shielded from light again for 5 min. For the cycle assay, after the cells were incubated with the conjugates for 24 h, the medium was collected into centrifuge tubes, and the cells underwent digestion with trypsin in each well. Following centrifugation at 1500 r for 5 min, the supernatant was discarded. Thereafter, EDTA-Na_2_ and RNAase were added and the mixture was left for 5 min at room temperature. Subsequently, Triton X-100 and PI solutions were added and protected from light for 10 min. At the end of the incubation, cells were resuspended with pbs. Set the appropriate excitation wavelength (488 nm) and emission wavelength (530 nm for Annexin V-FITC, and 630 nm for PI) for testing by flow cytometry.

### 2.11 Pharmacokinetics

The standard curve of SGI 1776 in blank plasma was established firstly. Blank plasma (0.1 mL) was added to SGI 1776 standard solutions of different concentrations (0.5, 2.5, 5, 12.5, and 25 μg/mL) and vortex evenly. The mixed solution was extracted three times with acetonitrile, purged with nitrogen, and then dissolved in chromatographic pure acetonitrile (400 µL) for HPLC analysis. The conditions of HPLC are the same as that of in the release test experiment. The standard curve was established by plotting the peak area and concentration. Repeat each step three times. Healthy C57 mice (20 ± 2 g) were randomly divided into three groups, with three mice in each group. The mice were raised for a week in a warm and well-ventilated environment. After fasting for 1 day, the mice were administered by gavage. The dosages of both the commercially available SGI1776 and the SGI1776 conjugate are 10 mg/kg. PBS was used as the control. Blood samples (0.1 mL) of mice were collected at 1 h, 2 h, 4 h, 8 h, 12 h, 24 h, and 48 h after administration. The blood was immediately transferred to a centrifuge tube containing EDTA K2 anticoagulant and centrifuged at 3,000 rpm for 10 min. The centrifuged plasma sample was transferred into a 2 mL centrifuge tube, chromatographic pure acetonitrile (400 µL) was added, vortex for 5 min, and centrifuge at 12,000 rpm for 10 min. Finally, the supernatant was filtered through a 0.22 μm microporous membrane and analysed by HPLC.

### 2.12 *In vivo* fluorescence imaging

The experimental procedures and euthanasia carried out in this study followed the guidelines of the Animal Ethics and Welfare Committee of cuiying biomedical research centre of Lanzhou University Second Hospital. The CDX model was developed by utilizing 143b osteosarcoma cells. These cells were prepared by suspending them in 100 μL of PBS and combining them with an equal volume of Matrigel (Corning, 354234), a matrix that mimics the extracellular environment. The cell-Matrigel mixture was then injected under the skin of 6-week-old immune deficient nude mice, with each mouse receiving 3 × 10^6^ cells. The Cy5.5 labelled conjugate was dispersed in PBS with the aid of ultrasound to form a solution with a concentration of 2 mg/mL. Male nude mice with osteosarcoma (20 ± 2 g) were randomly divided into two groups (n = 3), which was purchased from the experimental animal center of Lanzhou University. One group received the conjugate via a tail vein injection (20 μL injection volume, at a dose of 20 g/kg). The control group was administered PBS. *In vivo* fluorescence imaging of mice was performed using a highly sensitive fluorescence imaging system designed specifically for small animals (VISQUE *In vivo* Smart-LF, Viewers). The imaging was collected at specific time points: at pre-injection, 6.0- and 24.0-h post-injection.

### 2.13 HE staining

The mice that underwent pharmacokinetic treatment 48 h later were sacrificed, and the main organs (heart, liver, spleen, lungs and kidneys) as well as the stomach and intestines were taken. After the organs were fixed in paraformaldehyde (4%) for 48 h, they were dehydrated with gradient alcohol (70%–100%), transparent with xylene, and finally embedded and sectioned. the slices were place in an oven (55 °C) for 4 h, then perform dewaxing, and finally gently wash the samples with distilled water. The slices were stained with hematoxylin (2–3 min), then soaked in hydrochloric acid alcohol (1%) for 3 s, soaked in blue promoting solution (1% ammonia water) for 10 s, and rinsed under running water for 1 min. Afterwards, the slices were stained with eosin for 1 min, rinsed with running water, dehydrated with gradient alcohol (70%–100%) for 2 min, and then immersed in xylene for 5 min. Finally, the slides were seal with neutral resin and observe them under a microscope. Histologic scoring of the pathology sections (Dai et al., 2014; Galeano et al., 2008; Klopfleisch, 2013).

### 2.14 HPLC conditions

The mobile phase selected for HPLC was methanol and acetic acid water (0.1%) in a ratio of 30:70, with a flow rate of 1 mL/min, a column temperature of 30 °C, and an injection volume of 10 µL. The amount of SGI-1776 was detected by measuring the ultraviolet absorption at 294 nm.

### 2.15 Statistical analysis

Data were repeated three times in parallel for each group, and data were presented as mean ± standard deviation. Data were statistically analyzed using analysis of variance (ANOVA) combined with Bonferroni’s multiple comparison test. p-values <0.05 were considered statistically significant.

## 3 Results and discussion

### 3.1 Preparation and characterization

**SCHEME 1:**
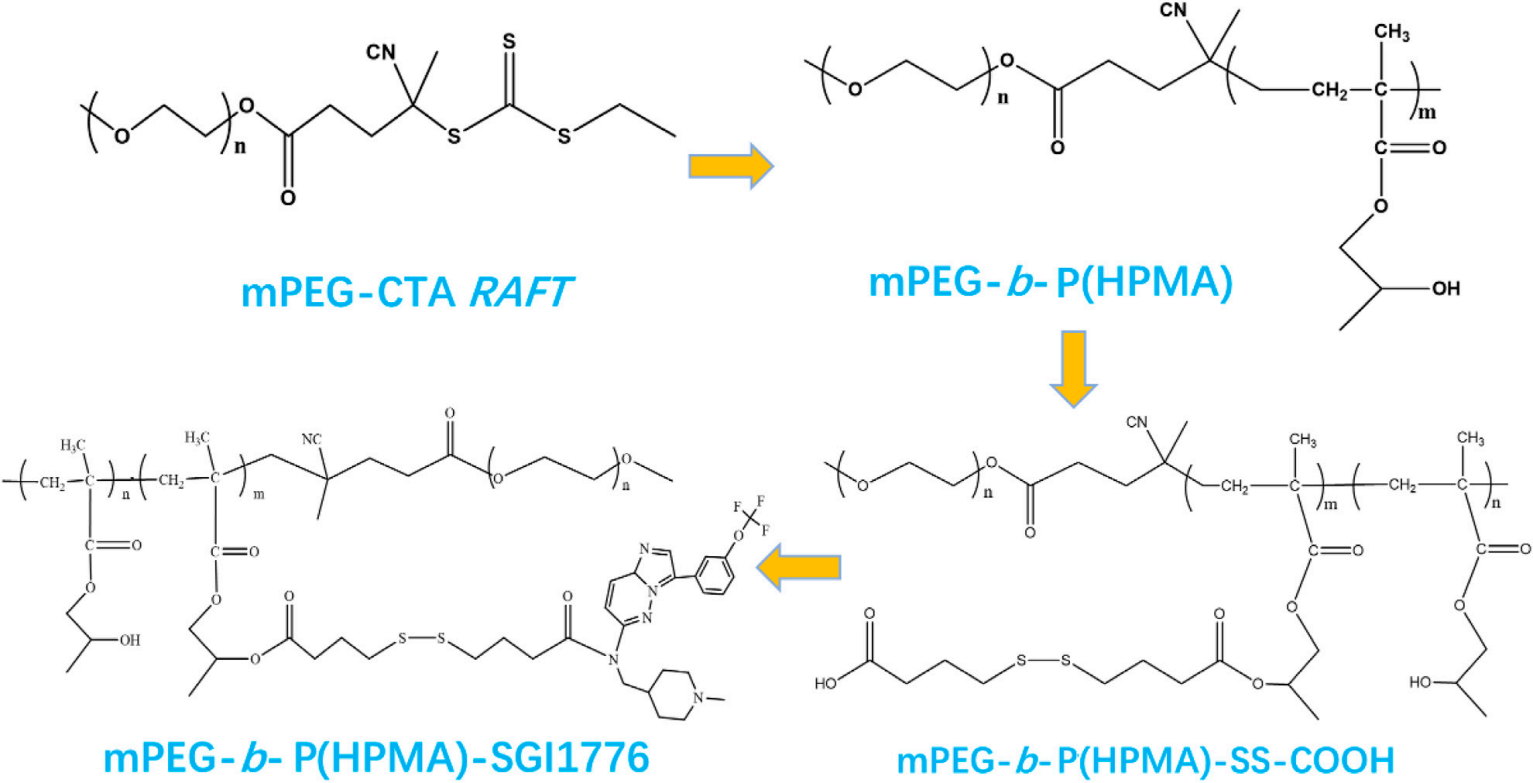
The fabrication of the mPEG-b-P(HPMA)-SGI1776 conjugate.

Scheme 1 illustrates the typical synthesis routes of the mPEG-*b*-P(HPMA)-SGI conjugate. The mPEG-*b*-P(HPMA) is fabricated via RAFT polymerization using mPEG-CTA as the macromolecular chain transfer agent. ^1^H NMR and MALDI-TOF spectrum of the mPEG-*b*-P(HPMA) precursor is exhibits in Supplementary Figures S1a, b. The chemical shift of hydrogen atoms in the characteristic group OCH_2_CH_2_ in mPEG is 3.65 ppm, which is labeled as a. The characteristic hydrogen atoms in the HPMA unit are labeled d and e with chemical shifts at 3.76 and 3.02 ppm, respectively. A series of peaks with molecular weight intervals of 144 are found in the MALDI-TOF spectrum, which corresponded exactly to the molecular weight of HPMA monomer. SGI-1776 is bound to the mPEG-*b*-P(HPMA) by linkers with disulfide bonds, which will facilitate the release of SGI1776 in the microenvironment of cancer cells with high glutathione content. The successful coupling of SGI1776 to the mPEG-b-P(HPMA) is confirmed by ^1^H NMR. All hydrogen atoms in free SGI1776 are numbered and the corresponding chemical shifts are summarized in Supplementary Figure S2a. The number of all the hydrogen atoms and the chemical shift correspond perfectly, proving that the structure of SGI1776 is correct. As for the mPEG-*b*-P(HPMA)-SGI conjugate (Supplementary Figure Sb), the signal of the -O-CH_2_CH_2_- in the PEG chain is found at 3.60 ppm. The signal of -O-CH_2_-CH- and the polymer chain in HPMA groups appear at 3.02 and 0.8–2.0 ppm. Excitingly, the signal of the SGI1776 group in the macromolecular prodrug appears between 7.0–8.5 ppm, indicating that the drug group is successfully linked to the polymer. More importantly, owing to the presence of fluorine atoms in SGI, the ^19^F NMR signals of SGI1776 and mPEG-*b*-P(HPMA)-SGI in specific mass are collected, further confirming the correctness of the structure (Supplementary Figure S2c). Subsequently, the semi-quantitatively assess the drug content within mPEG-*b*-P(HPMA)-SGI using the ratio of 19F NMR peak areas. As shown in Supplementary Figure S3. The fluorine atom signal appears at −56.5 ppm in SGI1776 and the conjugate. The chemical shift moves towards the high field after coupling to the polymer, which may be caused by the large amount of PEG wrapping. There are no aromatic ring groups and fluorine atomic in the initial reactants (mPEG-RAFT) and intermediates during preparation process (mPEG-*b*-P(HPMA) and mPEG-*b*-P(HPMA)-SS-COOH). When the reaction of SGI1776 and mPEG-*b*-P(HPMA)-SS-COOH is successful, benzene rings and fluorine atomic groups will appear in the mPEG-*b*-P(HPMA)-SGI conjugate formed, and then signals will appear at the corresponding chemical shifts in the ^1^H NMR and ^19^F NMR spectra. The integral-area-ratio calculation found that the mass fraction of drugs in conjugate is about 22%. Drug loading is an important evaluation index of nanomedicine (Shen et al., 2017). Too low a load can lead to an increase in the number of dosing times, which can increase the risk of infection. However, too high drug loading may affect the stability of nanomedicine. In this study, the payload of SGI1776 is regulated at 22%, which not only ensured the stability of the conjugate, but also ensured the therapeutic efficiency of the drug.

The molecular weight of the target conjugate is measured by MALDI-TOF. The results are displayed in Supplementary Figure S4. It can be seen that the molecular weight of the polymer shows a good positive distribution shape, and the central mass is about 5212. 1. This has a molecular weight approximately 2500 higher than that of the original mPEG. This is attributed to the polymerization of HPMA. The molecular weight values have an interval of 44, which is consistent with the molecular weight of the polymerization unit in PEG (Czaplewska et al., 2015). The results of mass spectrometry further prove the correctness of the conjugate. In general, the narrower the molecular weight distribution, the better the stability of nanomedicine and the higher the drug delivery efficiency (Choi and Han, 2018). However, because the molecular weight distribution of raw material PEG is difficult to control in industrial production, it is difficult to obtain accurate results on the effect of molecular weight distribution on drug delivery efficiency. We will focus on this question in future research.

The exact content of SGI in the polymer prodrug is further determined using UV spectrophotometry. Pure SGI1776 is dissolved in methanol at varying concentrations and ultraviolet absorption curves are recorded respectively (Supplementary Figure S5a). Despite an increase in absorbance intensity with increasing concentration, the maximum ultraviolet absorption wavelength at 210 nm for the pure SGI1776 solution unaffected by concentration variations. The absorbance of all solutions is adjusted to a range between 0.2 and 0.8 to minimize systematic errors during the testing process. The standard curve is obtained by plotting absorbance against concentration exhibits a strong linear relationship (Supplementary Figure S5b). The maximum ultraviolet absorption wavelength of the mPEG-*b*-P(HPMA)-SGI solution is identical to that of pure SGI1776, which may be attributed to the absence of other UV-absorbing functional groups in the carrier, mPEG-*b*-P(HPMA)-SS-COOH. The absorbance at the maximum absorption wavelength also increases with increasing concentration. Upon inputting the absorbance of the conjugate into the standard curve for calculation, the mass fraction of the drug in conjugate is approximately 24% (Supplementary Figure S5c). The results are in good agreement with those calculated by NMR. The FTIR is employed to further confirm the successful integration of the SGI functional group into the polymer matrix (Supplementary Figure S5d). The characteristic infrared vibration peaks of various functional groups in free SGI1776 are summarized in Supplementary Table S1. The characteristic vibration peaks of benzene ring and trifluoromethyl in pure SGI1776 are located at 2927, 2887 and 1220, 1150 cm^-1^. As for mPEG-*b*-P(HPMA)-SS-COOH, the peaks located at 3483 and 2882 cm^-1^ are respectively related to the stretching vibrations of O–H and C–H (Xu W. et al., 2023). The characteristic peak at 1728 cm^-1^ is assigned to the vibration of carbonyl groups. The stretching vibrations of C-N and C-O are observed at 1317 cm^-1^ and 1035 cm^-1^, respectively. There is no the benzene ring and trifluoromethyl vibration absorption peak in the spectrum of mPEG-*b*-P(HPMA)-SS-COOH because the polymer does not contain those groups. In case of mPEG-*b*-P(HPMA)-SGI1776 conjugate, the characteristic vibration peaks of benzene ring and trifluoromethyl are observed at 2927, 2887 and 1220, 1150 cm^-1^. This indicates that SGI1776 is indeed successfully attached to mPEG-*b*-P(HPMA)-SS-COOH.

The TGA and DTG curves of the conjugate is shown in Supplementary Figure S5e. For the carrier mPEG-*b*-P(HPMA)-SS-COOH, a sharp and gently weightlessness process is observed in the temperature range of 200 °C–420 °C and 420 °C–550 °C with a mass loss of about 90% and 10%, respectively. This process is basically consistent with the weightlessness process of the conjugate. The process of weightlessness reflects two obvious exothermic peaks on the DTG curve. The center of the first exothermic peak of both samples is located at 348 °C, which corresponds to the first stage of thermogravimetry. The center of the second exothermal peak of the conjugate and the carrier is located at 450 °C and 500 °C, respectively, which is caused by the introduction of SGI1776. Although the application temperature of the conjugate is under physiological temperature conditions, the drug may face extreme high temperature in some special cases during storage, so it is very important to clarify the thermal stability properties of the drug (Di Martino et al., 2017).

**FIGURE 1:**
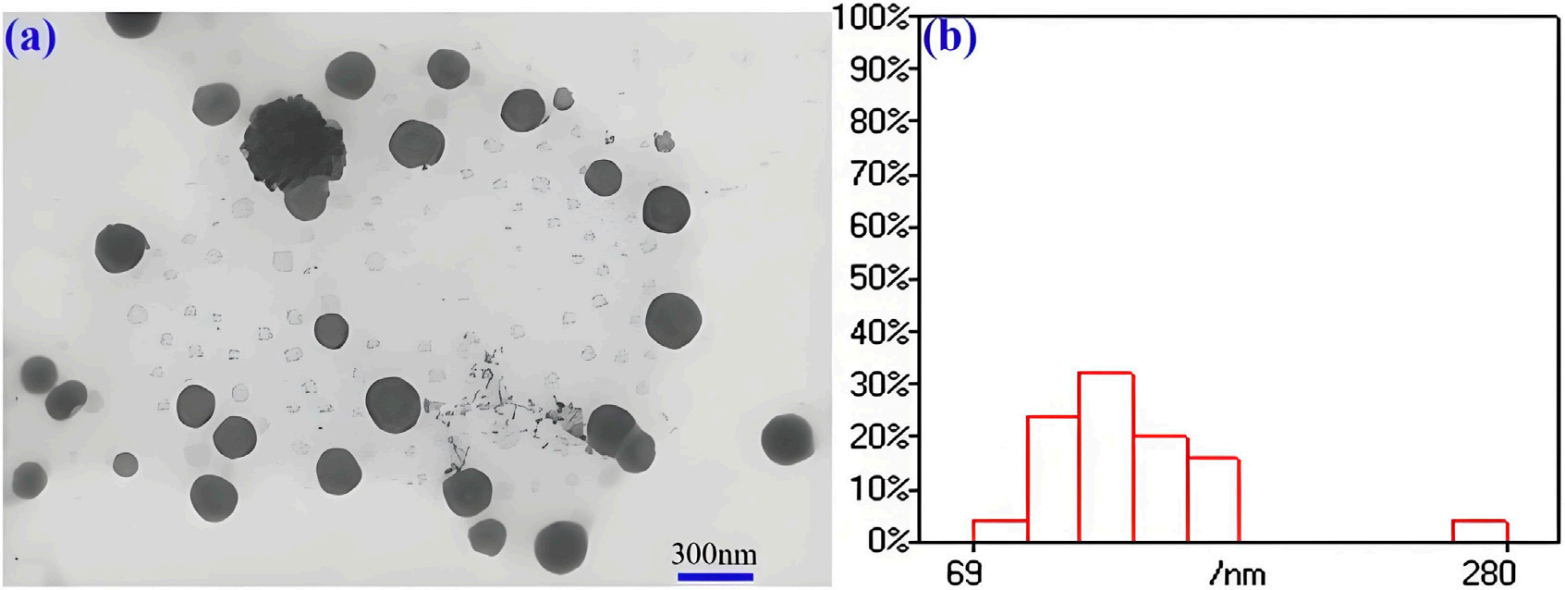
TEM images **(a)** and size statistics **(b)** of mPEG-*b*-P(HPMA)-SGI conjugate.

The particle size and surface potential of conjugate are tested for 5 consecutive days (Supplementary Figure S5f). The average particle sizes and PDI of the conjugate is 247 nm and 0.37 for the first day, respectively, suggesting good dispersion performance. As the time extends to the fifth day, the particle size and PDI increase up to 307 nm and 0.46. It can be seen that the particle sizes and PDI of the conjugate show insignificantly change though them are immersed in PBS solution for 120 h in PBS. The Zeta potential values of the conjugated material are continuously measured over a period of 5 days. The Zeta potential values on the first day and the fifth day are −5.46 mV and −5.82 mV, respectively. Although there are obvious changes in particle size, PDI and potential during five consecutive days of observation, the drugs in this study are connected to the carrier through chemical bonds, which is different from most drugs that were only physically encapsulated, so the observed changes had little impact on drug storage and *in vivo* application. This self-assembly property imparts the conjugate with enhanced permeability and retention (EPR) effect, thereby facilitating improved anti-tumor therapeutic efficacy (Guo et al., 2023). Due to the amphiphilic nature of the conjugate, it can self-assemble into nanoscale particles with micellar structures in aqueous solutions, as depicted in Figure 1a. It can be found a large number of spherical particles with a diameter of about 100–300 nm is observed. Nanoparticles within the field of view are uniformly dispersed without evident aggregation. the statistical analysis of particle size for 100 particles is shown in Figure 1b, it can be seen that a large number of particles are directly between 70–200 nm. These properties may have the potential lead to high antitumor efficacy via EPR effect. In additional, the conjugate exhibits excellent release profiles *in vitro* (Supplementary Figure S6a). Cumulative release amounts of SGI can be detected to be 52.9% and 2.8% at the concentration DTT of 10 and 0 μM at 24 h, respectively. The values are 76.5% and 4.9% as the time up to 96 h. This demonstrates that the redox sensitive character of the conjugate for the release of the drug results from the cleavage of the disulfide bond (Tan et al., 2023). In this work, the SGI1776 is connected to the polymer by disulfide bonds, which are reduced by two sulfhydryl groups in DTT or GSH to two independent hydrogen sulfide bonds, resulting in the release of SGI1776 from the conjugate. The reaction rate of the disulfide bond with glutathione/DTT is affected by many factors, such as temperature, reactant concentration, the presence of catalyst, and the regulation of reaction conditions. Because the concentration of glutathione in tumors is 7–10 times higher than in normal tissues, the release rates of SGI1776 in this conjugate is faster in tumor cells than in normal cells. The SGI1776 in the released solution is verified by HPLC (Supplementary Figure S6b). With the extension of time, the peak area of SGI1776 with a retention time of 8.65 min gradually increases. This indicates that the conjugate can release SGI1776 into the environment under the stimulation of DTT.

### 3.2 Cell activity assay

The hemolytic results of mPEG-*b*-P(HPMA)-SGI1776 conjugate are shown in Supplementary Figures S6c, d. Interestingly, even when the concentration is raised to 1.0 mg/L, the hemolysis rate is about 0.24%, which is well below the 5% hemolysis rate threshold for severe hematotoxicity (Soto et al., 2023). This may be related to the core-shell structure formed by self-assembly, and the low toxicity and good biocompatibility of the PEG outer layer reduces the damage of drugs to blood cells. A comparison of hemolysis rates of various polymer-drug conjugate is shown in Supplementary Table S2. Compared with conjugates such as PEG-curcumin/mefalan/chlorambucil/docetaxel, it can be seen that the conjugate prepared in this study showed lower hemolysis.

The inhibitory activity of mPEG-*b*-P(HPMA)-SGI1776 conjugate against 143b cells is assess using MTT colorimetry method (Supplementary Figure S6e). A trend of cell survival dependent on concentration is observed for both free SGI and conjugate. The IC_50_ of pure SGI1776 is about 8.9 μg/mL at incubation time of 24 h. As to the conjugate, The IC_50_ value is about 18.8 μg/mL, which is close to the value of pure SGI1776. The potential reason for the delay of drug action by conjugate of different sizes is that the size affects the uptake of conjugate by cells. The uptake of large-sized conjugate by cells is more difficult than that of small-sized, so larger conjugate will take longer to enter the cell and the drug will take longer to work (Feng and Tong, 2016). In addition, in the process of setting the drug concentration, the conjugate is calculated by converting the content of SGI1776 to the pure SGI1776 equivalent concentration. Therefore, the concentration of SGI1776 in the conjugate added in the MTT experiment is the same as the concentration of pure SGI1776. It can be seen from the results that for pure SGI1776, even at low concentration, the cell survival rate decreased significantly with the increase of concentration. For the conjugated compound, the cell survival rate did not change significantly with the increase of the concentration at low concentration, and the cell survival rate decreased significantly with the increase of the concentration to a certain value. This may be caused by the fact that the mPEG shell in the conjugate is obstructing the outward diffusion of the drug at low concentrations (Li et al., 2014).

**FIGURE 2:**
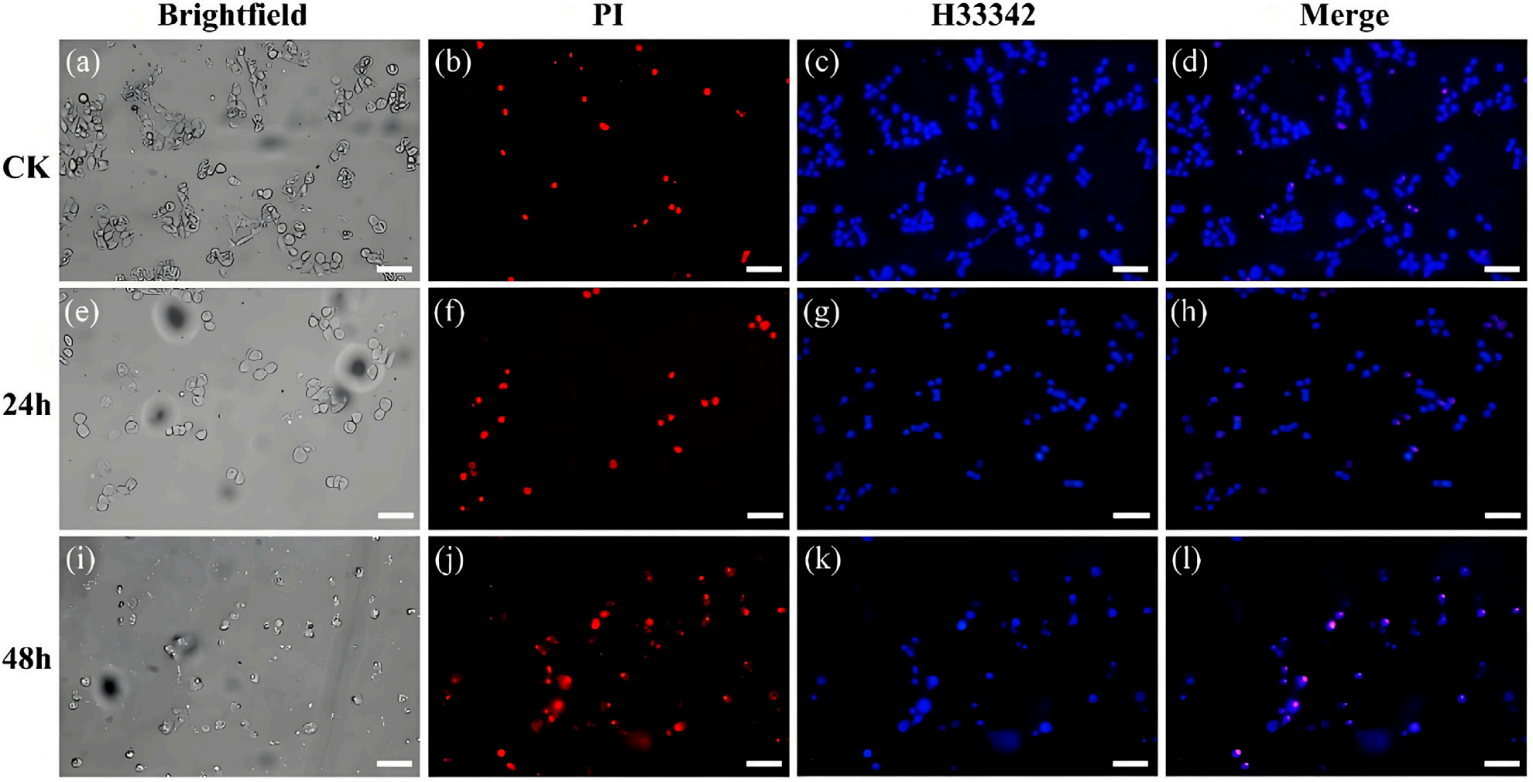
Bright-field and fluorescence images of cells stained with PI and Hoest33342 after treatment with the control group **(a–d)** and the conjugate for 24 h **(e–h)** and 48 h **(i–l)**, (Scale bar, 50 μm).

**FIGURE 3:**
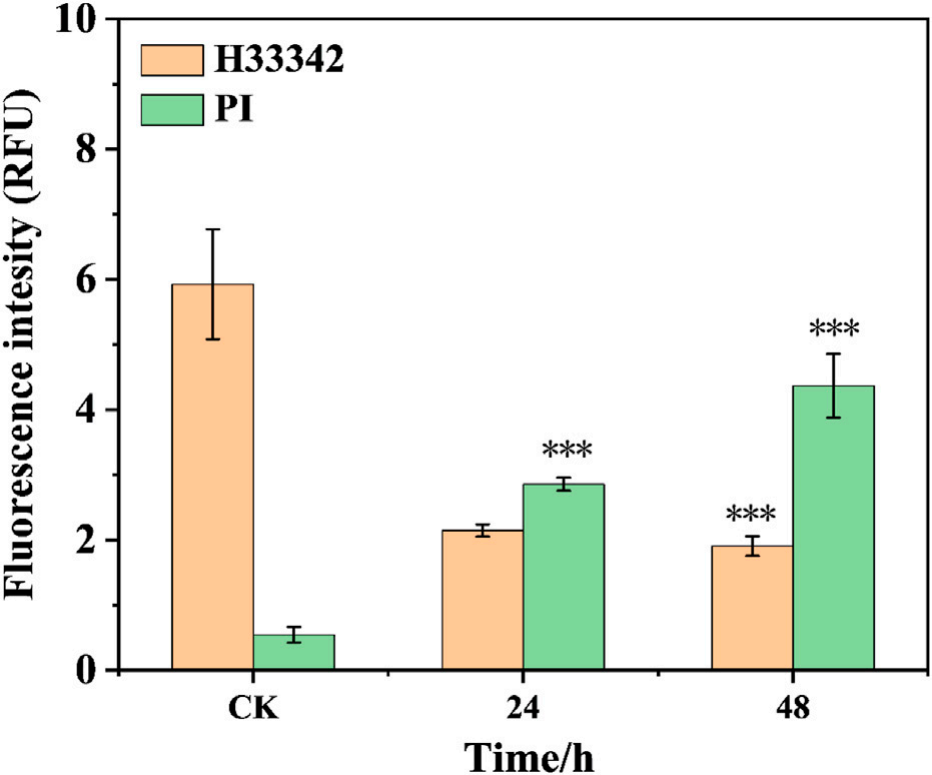
The fluorescence intensity of cells treated with the conjugate at different times (***p < 0.001, ****p < 0.0001).

The hoest33342 and PI staining method are employed to observe the effect of the conjugate on the morphology of 143b cells (Figure 2). The control group cells present normal circular or elliptical shapes, bright blue fluorescence, and imperceptible red fluorescence. Compares with the intact cells in the control group, the cells incubate with conjugate shows obvious chromatin condensation, fragmentation and other morphological changes. Combine with the apoptotic bodies observed, this indicates that the cells are in the apoptotic stage (Zhang X. et al., 2018). The degree of morphological change of cells, such as sphericity, atrophy and folding, deepened with the increase of incubation time, and the adhesion ability decreases significantly, and some cells even divide into small particles. Meanwhile, the pronounced blue fluorescence and the limited red fluorescence is observed, suggest that the conjugates are associated with the initial stages of apoptosis. Their fluorescence intensity was quantified (Figure 3). The results showed that after staining with PI, the red fluorescence intensity of the cells treated with this conjugate increased significantly by 5.6 and 8.6 times at 24 h and 48 h compared with the control.

**FIGURE 4:**
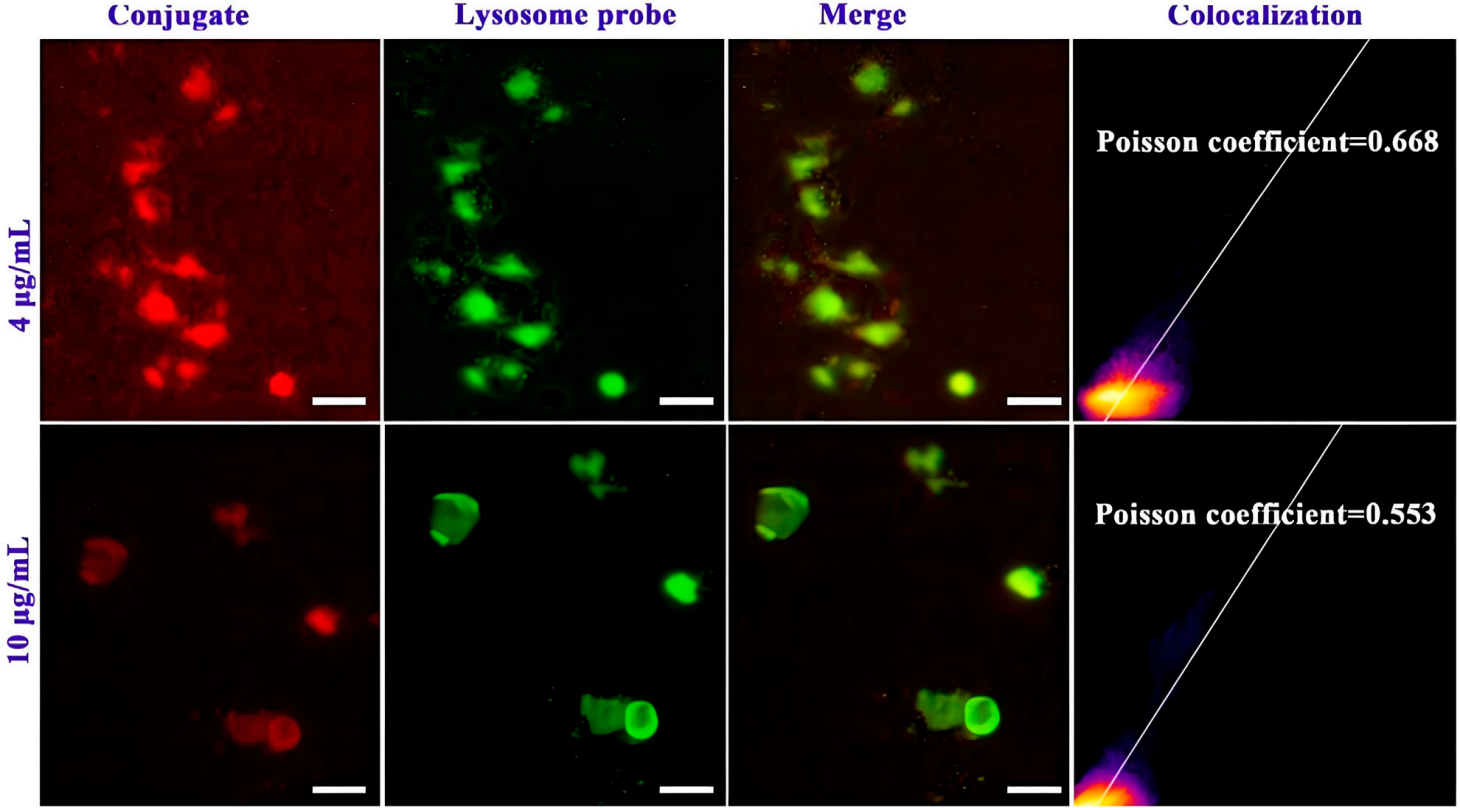
Lysosomal escape of Cy5.5 labelled mPEG-*b*-P(HPMA)-SGI conjugate, (Scale bar, 50 μm).

Subsequently, the ability of the conjugate to escape lysosome degradation is studied by using fluorescence image co-localization technique (Lee et al., 2022; Zhou et al., 2023). The cells are incubated with Cy5.5-labelled conjugate and lysosomal specific fluorescent dyes, and then confocal laser microscopy is used to collect the distribution of two kinds of fluorescence in the cells, and analyse the degree of fluorescence fusion. As shown in Figure 4, the red fluorescence of the conjugate is clearly separated from the green fluorescence of the lysosome. The merge of the both fluorescence shows a lighter yellow color, indicating that the conjugate can escape from lysosome capture and exhibit good lysosomal escape function, which helps to reduce the destruction of the drug in lysosome site and promote drug delivery to the nucleus. The Pearson’s correlation coefficient (R_r)_ values of the conjugate at 20 and 50 μg/mL are detected to be 0.668 and 0.533, respectively. This further quantified the excellent lysosomal escape ability of the conjugate.

**FIGURE 5:**
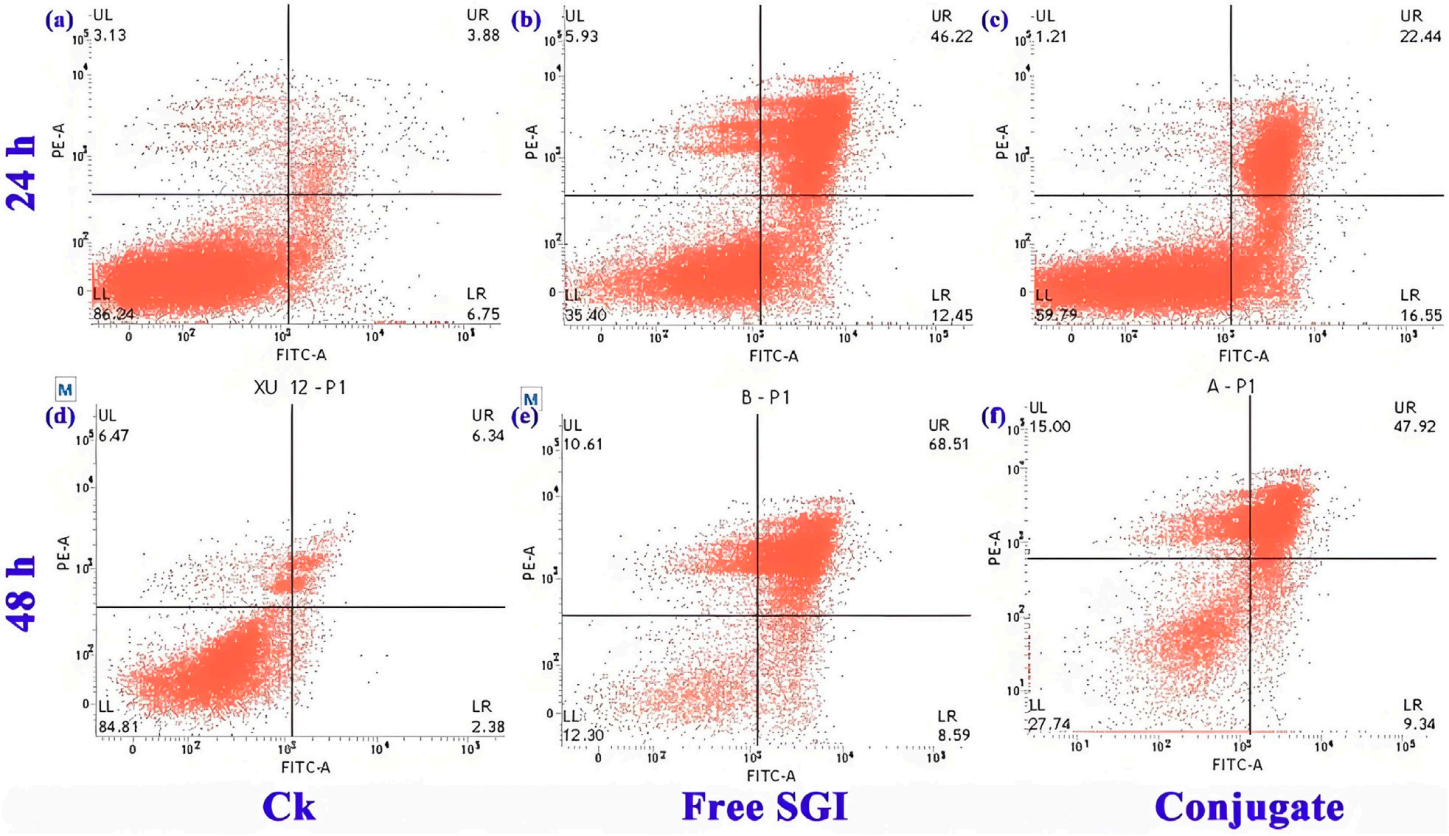
Flow cytometric analysis of 143b cells after incubation with PBS **(a,d)**, 10.5 mg L-1 free SGI1776 **(b,e)** and mPEG-b-P(HPMA)-SGI1776 conjugate **(c,f)** for 24 and 48 h.

**FIGURE 6:**
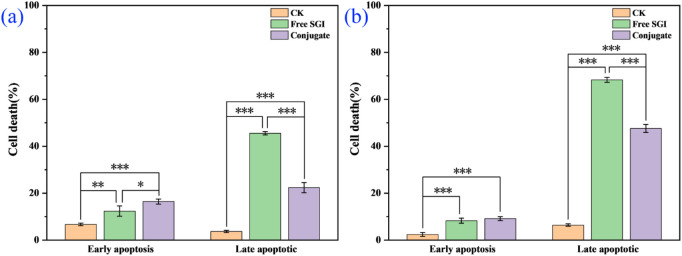
Control, free SGI and coupler in the proportion of apoptotic cells at 24 h **(a)** and 48 h **(b)**, respectively. (*p < 0.05, **p < 0.01, ***p < 0.001).

The effect of the conjugate on the division cycle of 143b cells is measured by flow cytometry (Ren et al., 2024). The details proportion of G0/G1, G2/M and S phase for the control, free SGI and conjugate are exhibit in Supplementary Figure S7 and Supplementary Table S3. The proportion of S phases for the PBS control, free SGI and conjugate is about 24.63%, 37.04%, and 36.44%. it can be found that the proportion of cells in the S phase in the drug-treated group was nearly 20% higher than that in the control group. The results show that the division cycle of 143b cells was blocked in S phase after drug treatment. The proportion of cells treated with the same concentration of free SGI and conjugation is basically the same in the three division stages, indicating that after 24 h of action, the conjugation has the same effect on cells as the free drug. The apoptotic rate of the 143b cells treated with free SGI and conjugate are examined by Annexin V/PI staining. The cellular density plots (Figure 5; Supplementary Table S4) shows the early apoptosis rates of the control, free-SGI, and conjugate treatments are 6.75, 12.41, and 16.55 after 24 h of drug treatment, respectively. The values of late apoptosis were 3.88, 46.22, and 22.44 (Figure 6). This indicates that pure SGI induced apoptosis in close to 50% of cells after 48 h of treatment. The apoptosis rate for conjugates is lower than that of free SGI. The reason may be that SGI1776 in the conjugates is wrapped in the inside of the nanoparticles and requires a certain concentration of GSH to release slowly and play a role. With the extension of time, a large number of active molecules are released, so the proportion of apoptosis increases with the extension of time. This GSH-responsive conjugate is able to release SGI1776 slowly, which facilitates less frequent dosing. These results further suggest that the cytotoxicity of the conjugate to cancer cells is mediated by inducing cellular apoptosis.

### 3.3 Pharmacokinetics and *in vivo* fluorescence imaging

**FIGURE 7:**
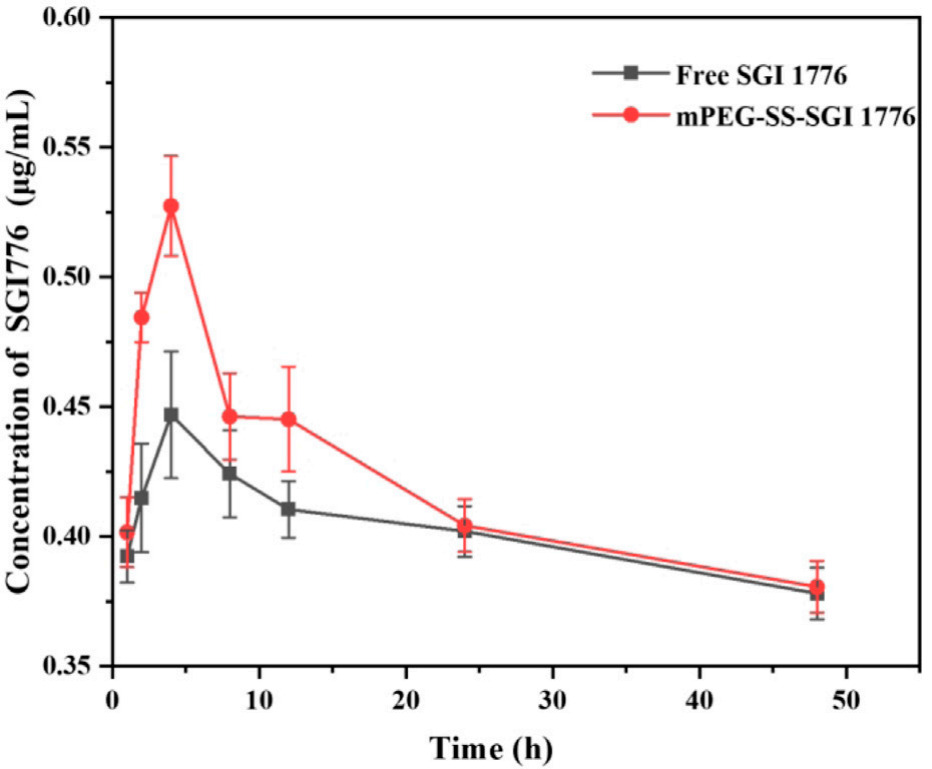
Concentration of SGI1776 in plasma collected from healthy mice following single oral administration of free SGI1776 and conjugate (10 mg/kg). Data represent mean ± standard deviation (n = 3).

Furthermore, the effect of this conjugate on the circulation time of SGI1776 *in vivo* is verified through pharmacokinetic studies. The liquid chromatograms and standard curves of SGI1776 in blank plasma are shown in Supplementary Figures S8a, b. It can be seen that there is a good linear correlation between the peak area and the concentration. The correlation coefficient reaches 0.9995. The liquid chromatograms of blood collected at different times after the administration of pure SGI1776 and the conjugate are summarized in Supplementary Figures S8c, d. The retention time of SGI1776 in the plasma of the two groups animals is almost the same as that of the standard substance. The peak area increases first increases and then decreases gradually with the extension of blood collection time. The pharmacokinetic curve and parameters is shown in Figure 7 and Supplementary Table S5. After the oral administration of a single dose of free SGI1776 solution and the conjugate, the peak plasma levels is attained at 4 ± 0 and 3.33 ± 1.15 h, respectively. The maximum plasma concentration is detected to be 0.45 ± 0.018 and 0.52 ± 0.092 μg/mL for free SGI1776 and the conjugate. The increase in C_max_ indicates that this responsive conjugate enhances the delivery capacity to SGI1776. The elimination half-life (T_1/2_) of the conjugate is observed at 19.21 ± 1.21 h, which is significantly longer than that of the free SGI1776 at 18.31 ± 2.31 h. The prolonged duration of the conjugate *in vivo* is conducive to the continuous therapeutic effect of the drug. More important, the AUC_0-48_ values of the free SGI776 and the conjugate group are 80.83 ± 2.34 and 131.17 ± 1.79 h*µg/mL, respectively. The marked enhances in AUC_0-48_ suggests that the conjugate enhances the bioavailability of SGI1776 (Su et al., 2023).

**FIGURE 8:**
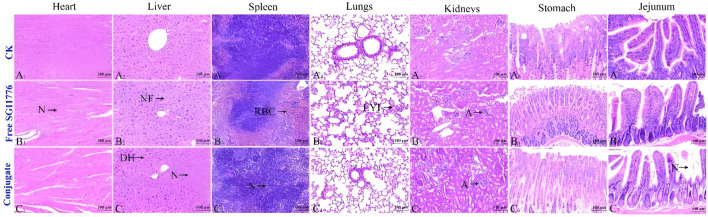
Healthy mice are injected with PBS, free SGI1776, and conjugate at a single dosage of 10 mg SGI1776/kg of animal body weight. H&E stained histological sections. Heart (A1-C1), liver (A2-C2), spleen (A3-C3), lung (A4-C4), kidney (A5-C5), stomach (A6-C6) and jejunum (A7-C7), (Scale bar, 100 μm).

**FIGURE 9:**
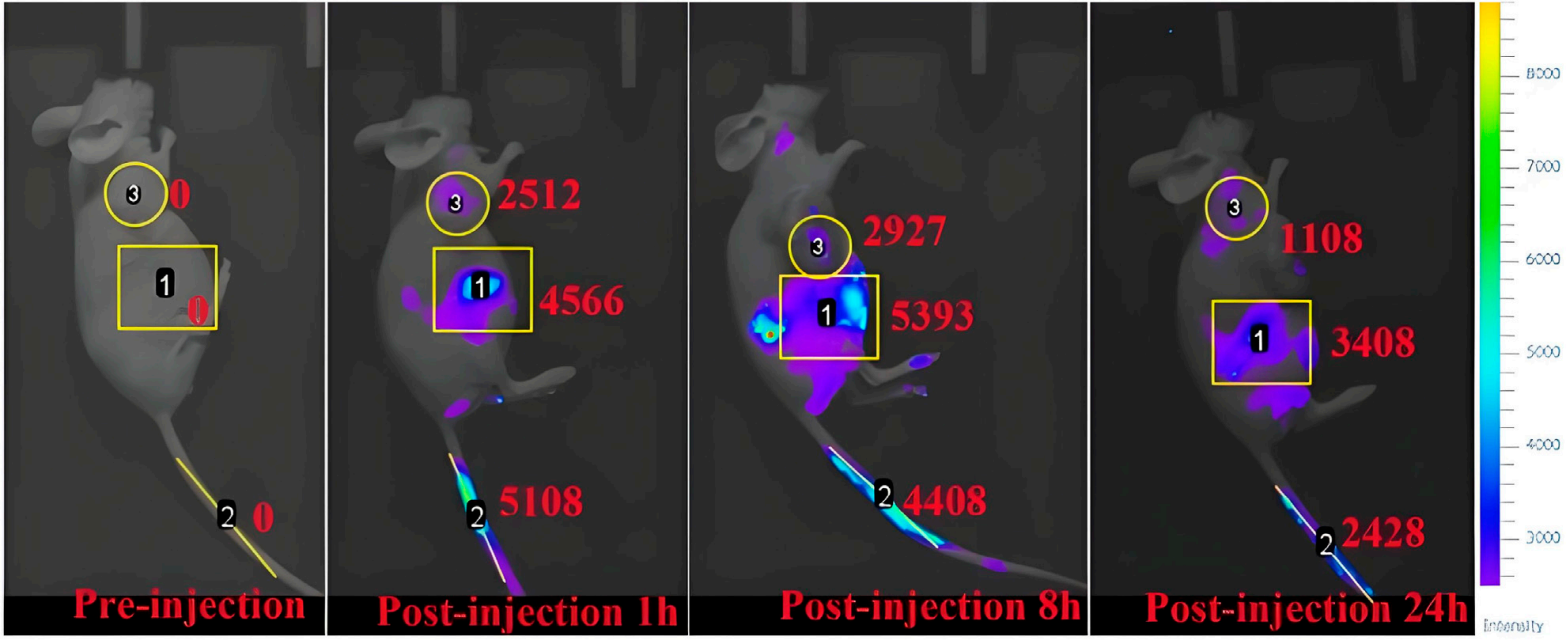
Fluorescence imaging of osteosarcoma-bearing mice after injection of mPEG-*b*-P(HPMA)-SGI1776 conjugate (20 μL injection volume, at a dose of 20 g/kg) at different time points.

**FIGURE 10:**
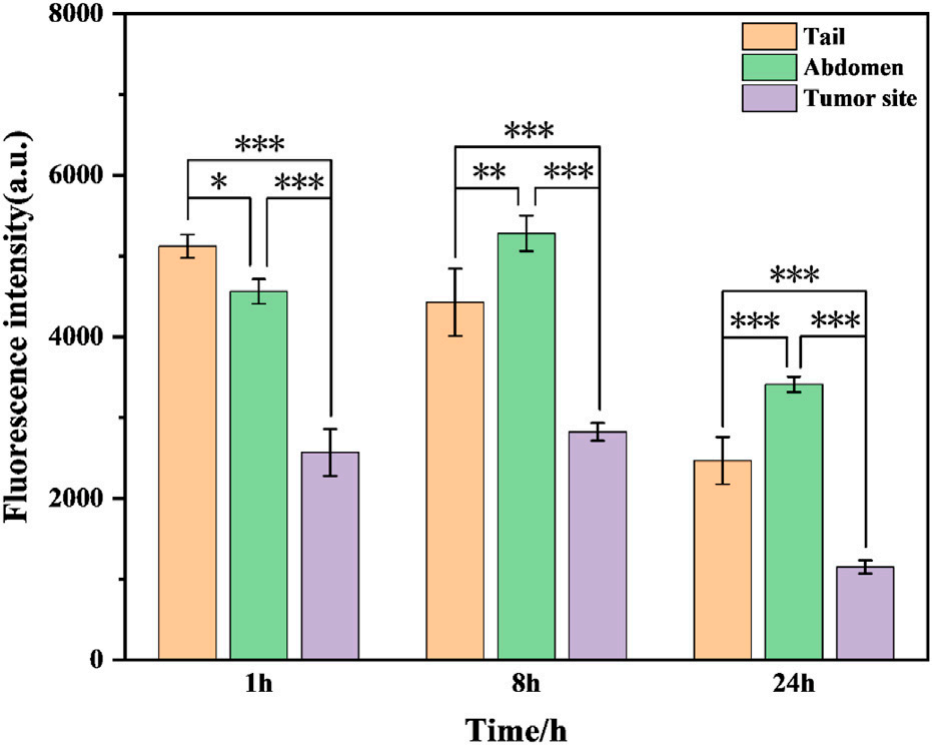
Fluorescence intensity of mPEG-b-P(HPMA)-SGI1776 coupling (injection volume of 20 μL, dose of 20 g/kg) at different time points of tail, abdomen and tumor site. (*p < 0.05, **p < 0.01, ***p < 0.001).

To further test the biosafety of the conjugate, the mice after pharmacokinetics were dissected, and the damage of the main organs was explored by H&E staining. The results are displayed in Figure 8. Local degeneration and necrosis of myocardial fibers occurred in B1. Focal necrosis of hepatocytes is observed in B2. In B3, splenic body degeneration, atrophy and reduced volume occurs, and a large number of red blood cells can be seen in the red marrow. Alveolar phrenic lymphocyte infiltration occurs in B4. Glomerular degeneration, atrophy and reduced volume are observed in B5. It can be seen that the free SGI1776 has caused relatively serious organ damage. For the conjugate, diffuse blister deformation and local necrotic foci can be observed in hepatocytes (C2). Degeneration and atrophy occurs in the splenic bodies and glomeruli (C3 and C4). There is necrosis and shedding of intestinal villi (C7). As indicated by the histologic score (Supplementary Figure S10). The conjugate significantly alleviates the damage of the drug to the heart and kidneys (Sreelaya et al., 2025). The distribution of conjugate *in vivo* is studied using fluorescently living imaging system (Figures 9, 10). There were obvious fluorescent signals in the abdomen, tail and around the tumor at post-injection 1.0 h. The relative fluorescence intensity in the tail, abdomen and tumor is about 5108, 4566, and 2512. The results showed that the nanoparticle is successfully injected into the body fluid circulation system of mice through the tail vein. The signal intensity in the tail is 1.1 and 2.0 times higher than that in the abdomen and around the tumor. This is due to the short injection time, which causes a large number of nanoparticles to accumulate at the injection site without time to enter the body fluid circulation. The images taken 8 h after injection show that the fluorescence areas of the tail and abdomen were significantly enlarged, and there are very obvious fluorescence signals in the tumor parts of the mice. The relative fluorescence intensity in the tail, abdomen and tumor is about 4408, 5396, and 2927. When the time is further extended to 24 h, the fluorescence intensity of the tail, tumor body and abdomen is significantly reduced than that of 8 h after injection. The results show that the fluorescence at the tumor site increased with the prolongation of drug circulation time, and the fluorescence intensity reached the maximum at the tumor site after 8 h of the injection via the tail vein. These results indicate that the conjugated compounds in this study exhibit passive targeting capabilities.

Aberrant expression of PIM kinases is observed in variety of tumors. Therefore, small molecule PIM inhibitors are a promising class of drugs for the treatment of solid tumors and hematological malignancies (Xu et al., 2014). SGI-1776 is one of the important PIM kinase inhibitors. Just as the Seo research reveals, when mice with lipopolysaccharide-induced bone loss or tumor-induced osteolysis were treated with SGI-1776, the bone loss is significantly ameliorated (Seo et al., 2025). In this work, the drug is designed and prepared as a conjugate with high GSH stimulation response in tumors, in order to improve the bioavailability of the drug and reduce side effects. This will provide many formulation options for advancing the clinical progress of drugs.

## 4 Conclusion

In summary, a novel GSH-responsive conjugate prodrug is facilely fabricated to delivery PIM kinase inhibitor SGI1776 for treatment of osteosarcoma. This conjugate exhibits typical spherical nanoparticles, and the loading of SGI1776 reaches 22%. Cumulative release rates can be detected to be 52.9% at the concentration DTT of 20 mM at 24 h. This conjugate exhibits a lower hemolysis rate and better lysosomal escape ability. It is more likely to induce apoptosis and can be blocked 143b cells in the S cycle phase. The results of *in vivo* studies indicate that this conjugate can significantly increase the utilization rate of the drug and reduce its side effects, which will be conducive to possible clinical studies in the future.

## Data Availability

The original contributions presented in the study are included in the article/Supplementary Material, further inquiries can be directed to the corresponding author.
