# Supplementary material for: Enhanced pharmacokinetic and therapeutic of GSH-responsive mPEG-b-P(HPMA)-SGI1776 conjugate for osteosarcoma
Source: Front Pharmacol. 2025 Oct 23;16:1676770. doi: 10.3389/fphar.2025.1676770 (PMC12588892; doi:10.3389/fphar.2025.1676770)
Supplement: Supplementary file 1 [file Supplementaryfile1.docx]

Supplementary Material

**Supplementary Table 1.** Infrared characteristic vibration peak of SGI1766

| **H** | **Wavenumber（cm^-1^）** | **Group** |
| --- | --- | --- |
| ν_N-H_ | 3246 | NH |
| ν_=C-H_ | 3050 | Benzene ring |
| ν_C-H_ | 2927,2887 | -CH_3_ |
| δ_C-H_ | 1384 | -CH_3_ |
| ν_C-H_ | 2788,2735,2675 | -CH_2_- |
| δ_C-H_ | 1485 | -CH_2_- |
| ν_C=N_ | 1624 | C=N |
| ν_C=C_ | 1587 | Benzene ring |
| ν_C-F_ | 1220, 1150 | -CF_3_ |

**Supplementary Table 2.** Comparison of hemolysis rates of different polymer-drug conjugate.

| Conjugate | Hemolysis rate | Concentration | Reference |
| --- | --- | --- | --- |
| PEG-curcumin | 0.09 ± 0.005 | 6.4 mg/mL | Dey et al., 2015 |
| Neomycin–phenolic | 0.75% | 100 μg/ml | Findlay et al., 2012 |
| PEG5000-melphalan | 48.8±1.5% | 32 µg/ml | Ajazuddin et al., 2013 |
| Chitosan-polylactic acid- curcumin | 1.5% | 100 μg/ml | Sutar and Telvekar, 2018 |
| mPEG-PLGA- Docetaxel | 3% | 0.6 mg/mL | Guo et al., 2016 |
| PEG-chlorambucil-biotin | 1.5% | 1.6mg/ml | Ghosh et al., 2024 |
| polymannose-doxorubicin | 3.0% | 0.2 mg/ml | Francis and Jayakrishnan, 2019 |
| Ki16425-PEG5k-CBZ | 2.5% | 0.1 mg/ml | Ozel et al., 2024 |
| mPEG-b-P(HPMA)-SGI1776 | 0.24% | 1 mg/ml | This work |

Ajazuddin, Alexander, A., Amarji, B. and Kanaujia, P. (2013). Synthesis, characterization and in vitro studies of pegylated melphalan conjugates. *Drug Dev. Ind. Pharm.* 39(7), 1053-1062. <https://doi.org/10.3109/03639045.2012.702346>.

Dey, S., Ambattu, L.A., Hari, P.R., Rekha, M.R. and Sreenivasan, K. (2015). Glutathione-bearing fluorescent polymer-curcumin conjugate enables simultaneous drug delivery and label-free cellular imaging. *Poly* 75, 25-33. <https://doi.org/10.1016/j.polymer.2015.08.020>.

Findlay, B., Zhanel, G.G. and Schweizer, F. (2012). Neomycin-phenolic conjugates: Polycationic amphiphiles with broad-spectrum antibacterial activity, low hemolytic activity and weak serum protein binding. *Bioorg. Med. Chem. Lett.* 22(4), 1499-1503. <https://doi.org/10.1016/j.bmcl.2012.01.025>.

Francis, A.P. and Jayakrishnan, A. (2019). Conjugating doxorubicin to polymannose: a new strategy for target specific delivery to lung cancer cells. *J. Biomater. Sci. Polym. Ed.* 30(16), 1471-1488. <https://doi.org/10.1080/09205063.2019.1646475>.

Ghosh, D., Khan, A., Bag, S., Mallick, A.I. and De, P. (2024). Dual stimuli-responsive biotinylated polymer-drug conjugate for dual drug delivery. *J. Mater. Chem. B* 12(45), 11826-11840. <https://doi.org/10.1039/d4tb01762e>.

Guo, Y., Zhang, P., Zhao, Q., Wang, K. and Luan, Y. (2016). Reduction-Sensitive Polymeric Micelles Based on Docetaxel-Polymer Conjugates Via Disulfide Linker for Efficient Cancer Therapy. *Macromol. Biosci.* 16(3), 420-431. <https://doi.org/10.1002/mabi.201500317>.

Ozel, B., Sanlier, S., Gunduz, C. and Gunel, N.S. (2024). Preparation of dual drug-loaded polymer nanoconjugate to enhance treatment efficacy for ovarian cancer cells. *Eur. J. Pharm. Biopharm.* 204. <https://doi.org/10.1016/j.ejpb.2024.114526>.

Sutar, Y.B. and Telvekar, V.N. (2018). Chitosan based copolymer-drug conjugate and its protein targeted polyelectrolyte complex nanoparticles to enhance the efficiency and specificity of low potency anticancer agent. *Mat. Sci. Eng. C-mater.* 92, 393-406. <https://doi.org/10.1016/j.msec.2018.07.001>.

**Supplementary Table 3.** Cell cycle proportion of G0/G1, G2/M and S phase for the control, free SGI and conjugate.

|  | G1 | G2 | S |
| --- | --- | --- | --- |
| Control | 64.85% | 10.52% | 24.63% |
| Free SGI | 40.38% | 22.58% | 37.04% |
| Conjugate | 42.33% | 21.23% | 36.44% |

**Supplementary Table 4.** Cell apoptosis proportion for the control, free SGI and conjugate.

|  | Annexin V-/PI-  (Living cell) | (Annexin V+/PI-)  early apoptosis | (Annexin V+/PI+  Late apoptotic | (Annexin V-/PI+)  early apoptosis |
| --- | --- | --- | --- | --- |
| Ck(24h) | 86.24 | 6.75 | 3.88 | 3.13 |
| Free SGI(24h) | 35.40 | 12.41 | 46.22 | 5.93 |
| Conjugate(24h) | 59.79 | 16.55 | 22.44 | 1.21 |
| Ck(48h) | 84.81 | 2.6 | 6.34 | 6.47 |
| Free SGI(48h) | 12.30 | 8.59 | 68.51 | 10.61 |
| Conjugate(48h) | 27.74 | 9.34 | 47.92 | 15.00 |

**Supplementary Table 5.** Pharmacokinetic parameters (Mean±SD) of the commercially available drug (SGI 776) and mPEG-SS-SGI 1776.

| Parameter | Unit | Value | |
| --- | --- | --- | --- |
|  |  | SGI 1776 | mPEG-SS -SGI 1776 |
| T_1/2 ab_ | h | 2.48±0.37 | 1.514±0.98* |
| T_1/2 el_ | h | 18.31±2.31 | 19.21±1.21* |
| T_max_ | h | 4±0 | 3.33±1.15 |
| C_max_ | µg/mL | 0.45±0.018 | 0.52±0.092** |
| AUC_0~t_ | h*µg/mL | 80.83±2.34 | 131.17±1.79** |
| MRT_0~t_ | h | 22.73±0.8788 | 21.99±1.165 |

T_1/2 ab_: absorption half-life; T_1/2 el_: elimination half-life; T_max_: time required for drug to reach maximum concentration; C_max_: maximum concentration; AUC_0~t_: the area under the curve; MRT_0~t_: mean residence time.( *p < 0.05, **p < 0.01)


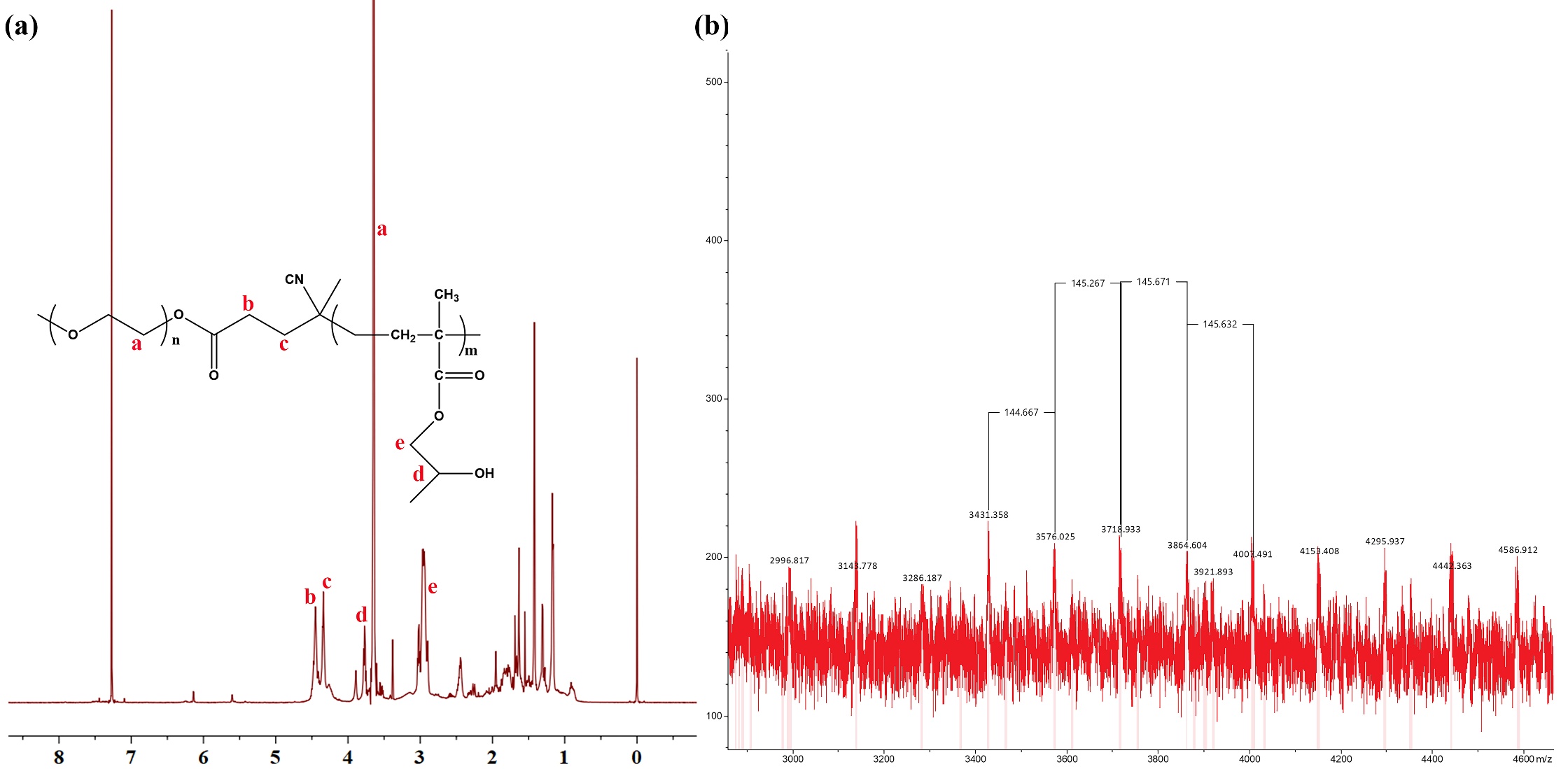


**Supplementary Figure 1.** ^1^H (a) and MALDI-TOF (b) spectra of mPEG-*b* P(HPMA) precursor.


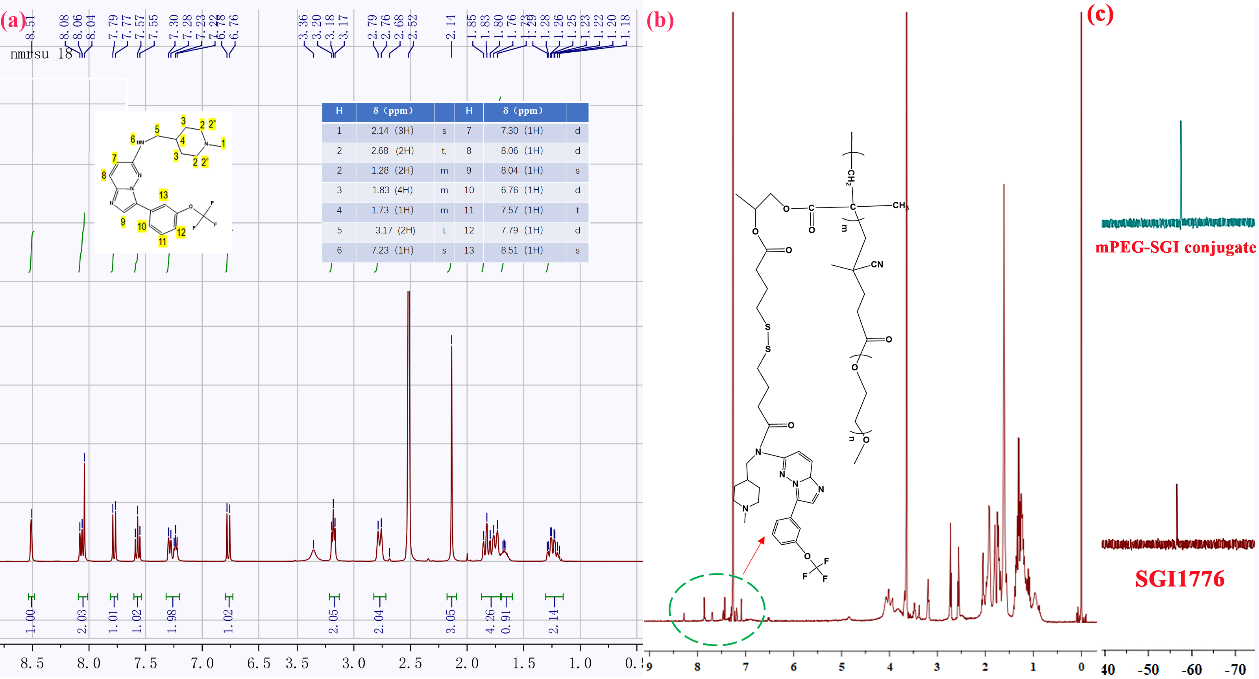


**Supplementary Figure 2.** The ^1^H (a and b) and ^19^F (c) NMR spectra of free SGI1776 and mPEG-*b* P(HPMA)-SGI conjugate.

**
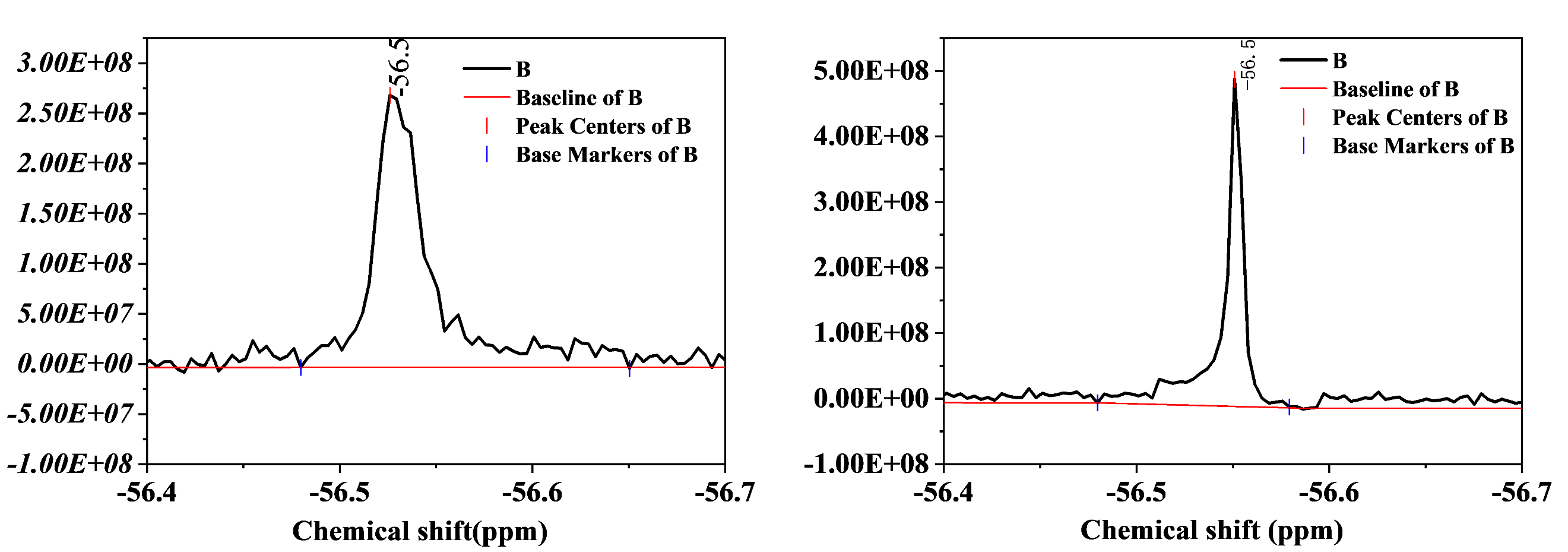
**

**Supplementary Figure 3.** Integral area ^19^F NMR spectra of free SGI1776 (a) and mPEG-*b*-P(HPMA)-SGI1776 conjugate (b).


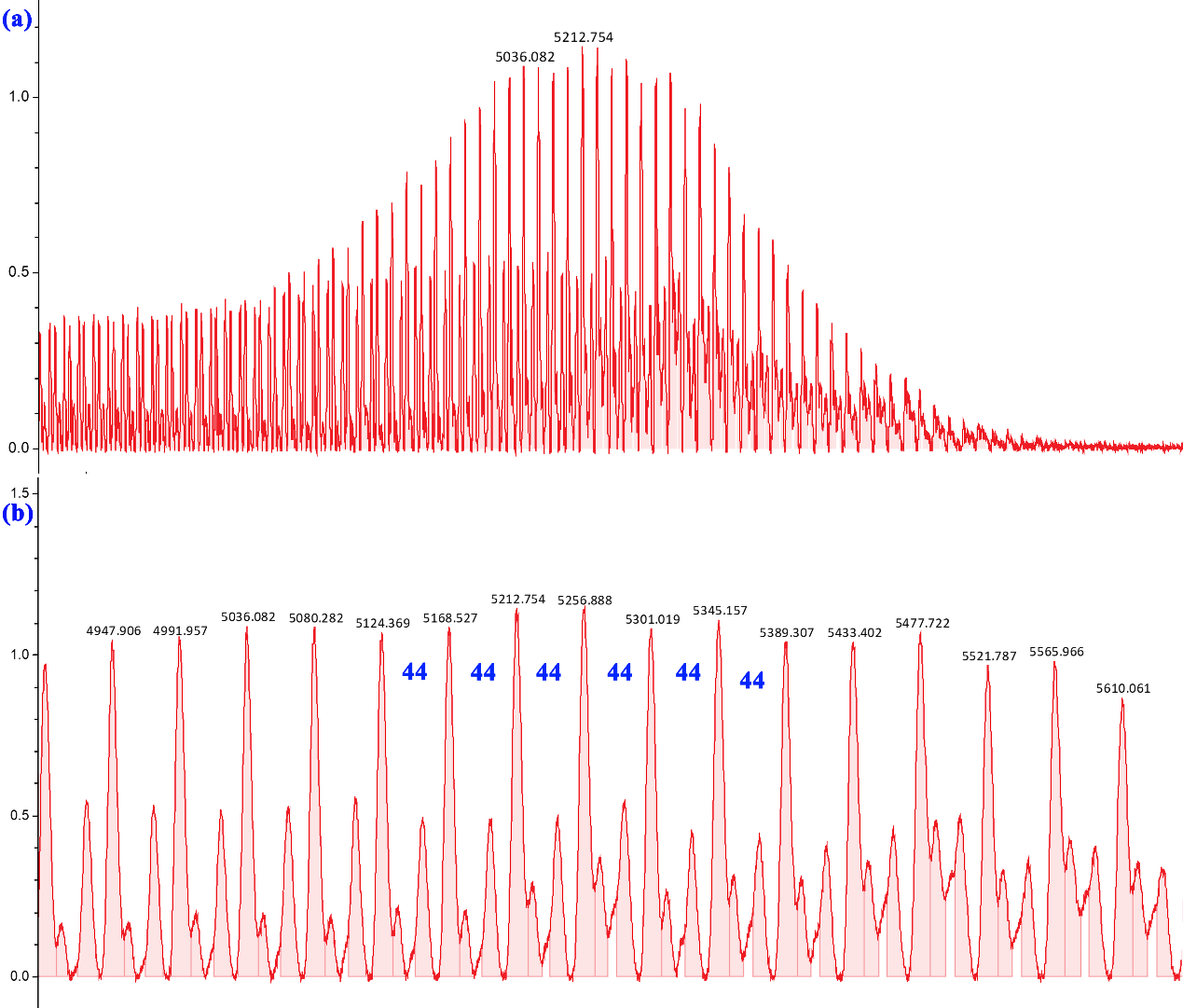


**Supplementary Figure 4.** MALDI-TOF spectra of the mPEG-*b*-P(HPMA)-SGI1776 conjugate.


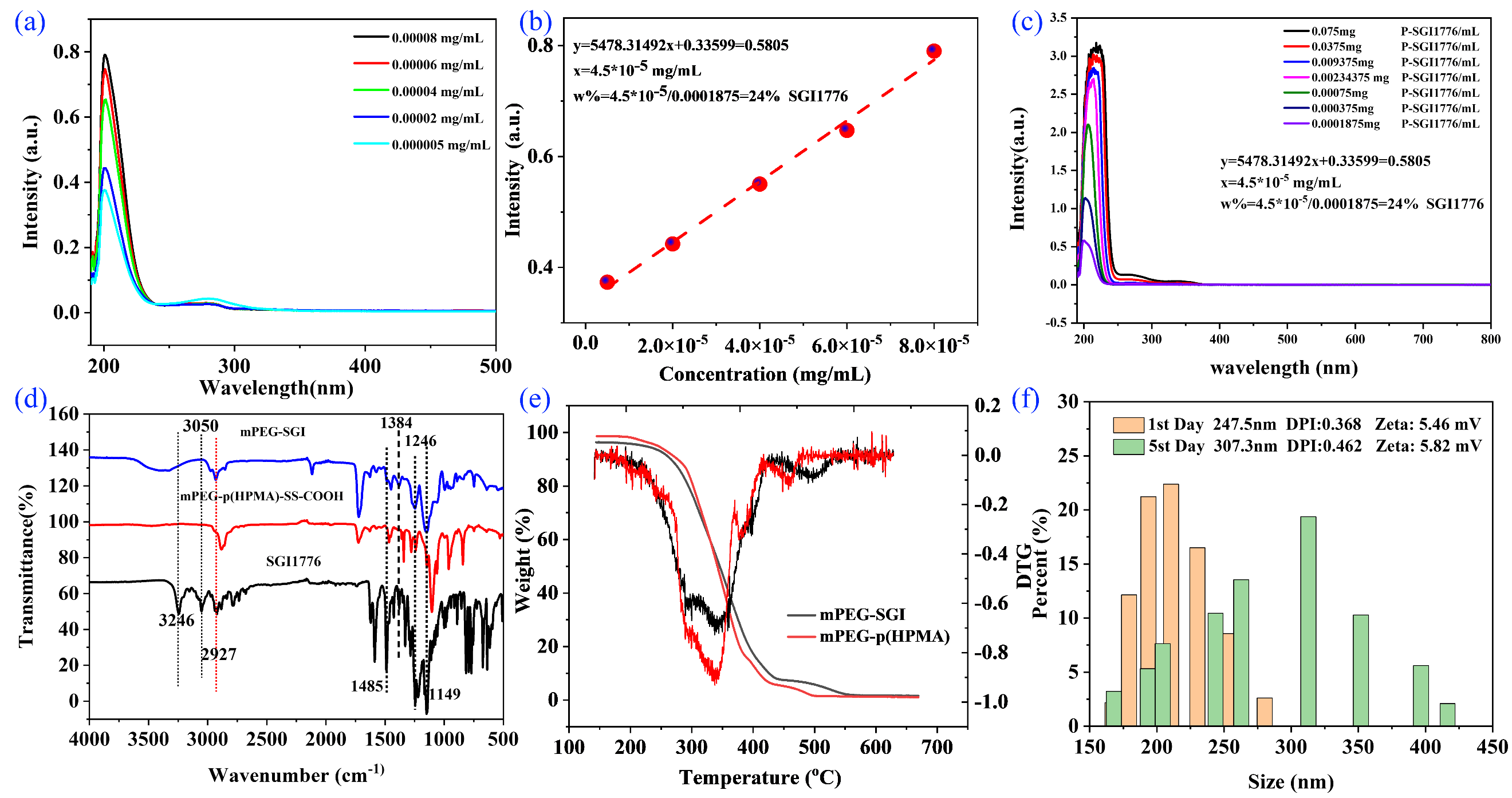


**Supplementary Figure 5.** UV curves (a) and calibration curve (b) of free SGI1776 with different concentration; the uv curves (c), FTIR spectra (d), TGA and DTG (e), Size (f) of mPEG-*b*-P(HPMA)-SGI1776 conjugate.


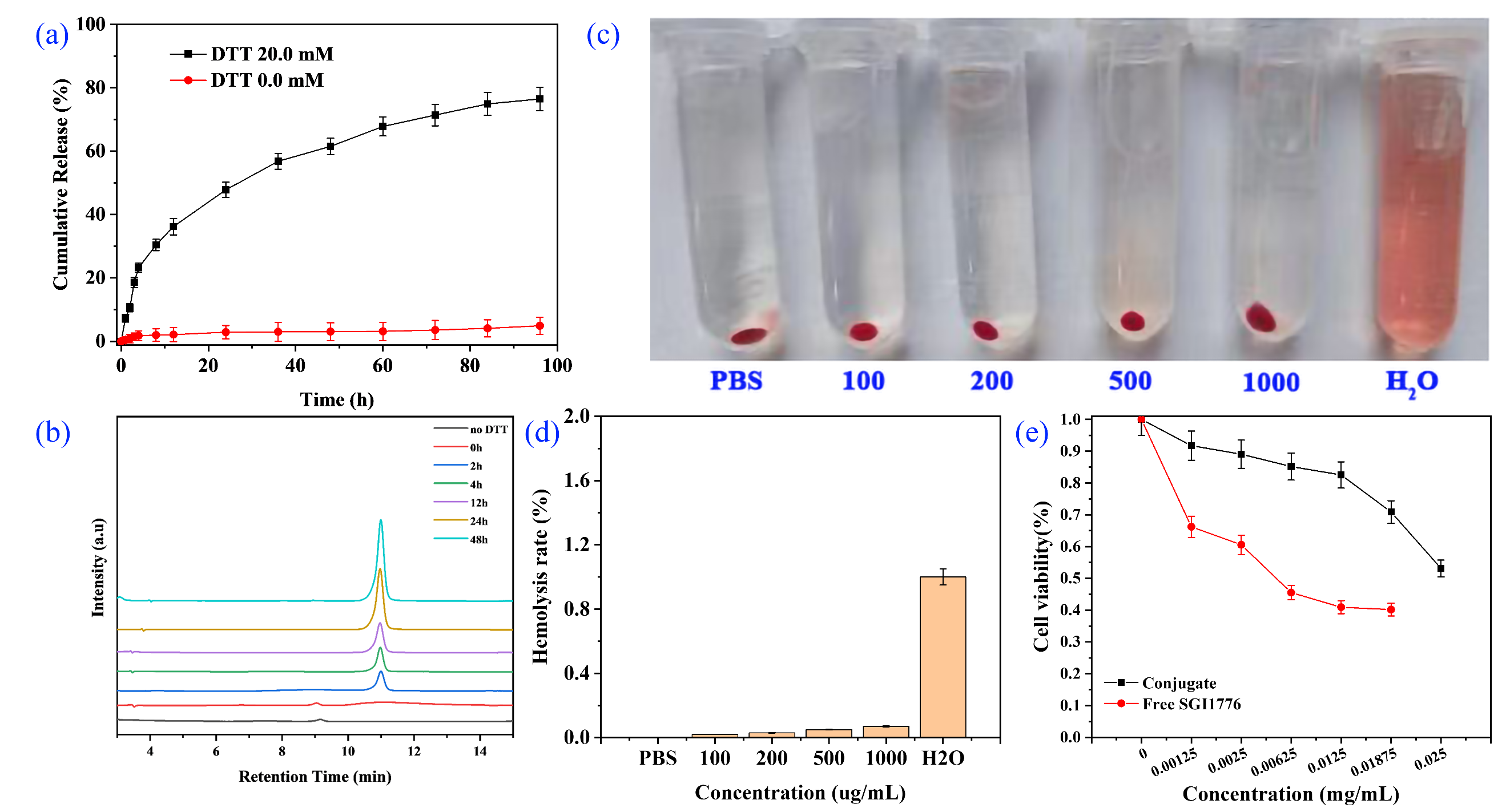


**Supplementary Figure 6.** Cumulative release profiles (a) and HPLC release profiles (b) of mPEG-SGI coupling at DTT concentrations of 0 and 20 mM. The hemolytic images (c) and Hemolysis rate (d) of mPEG-SGI conjugate; the MTT results (e) of the free SGI1776 and mPEG-*b*-P(HPMA)-SGI1776 conjugate.


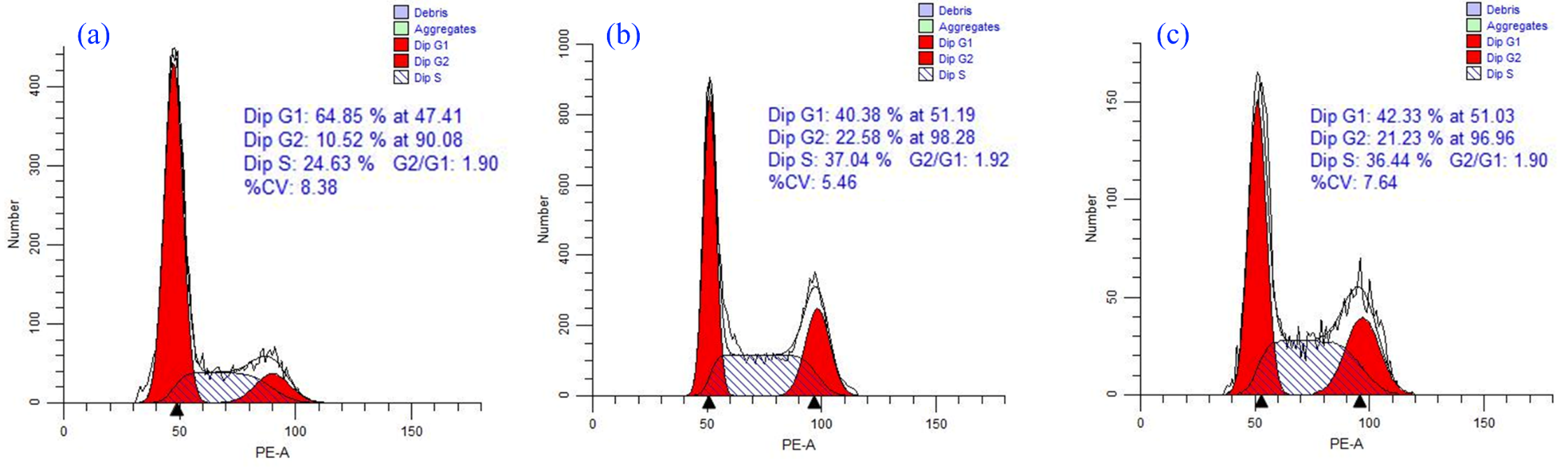


**Supplementary Figure 7.** The cycle data of 143b cells incubated with PBS (a), free SGI1776 (b) and mPEG-*b*-P(HPMA)-SGI1776 conjugate (c) at concentration of 10.5 mg L^-1^ for 24 h.


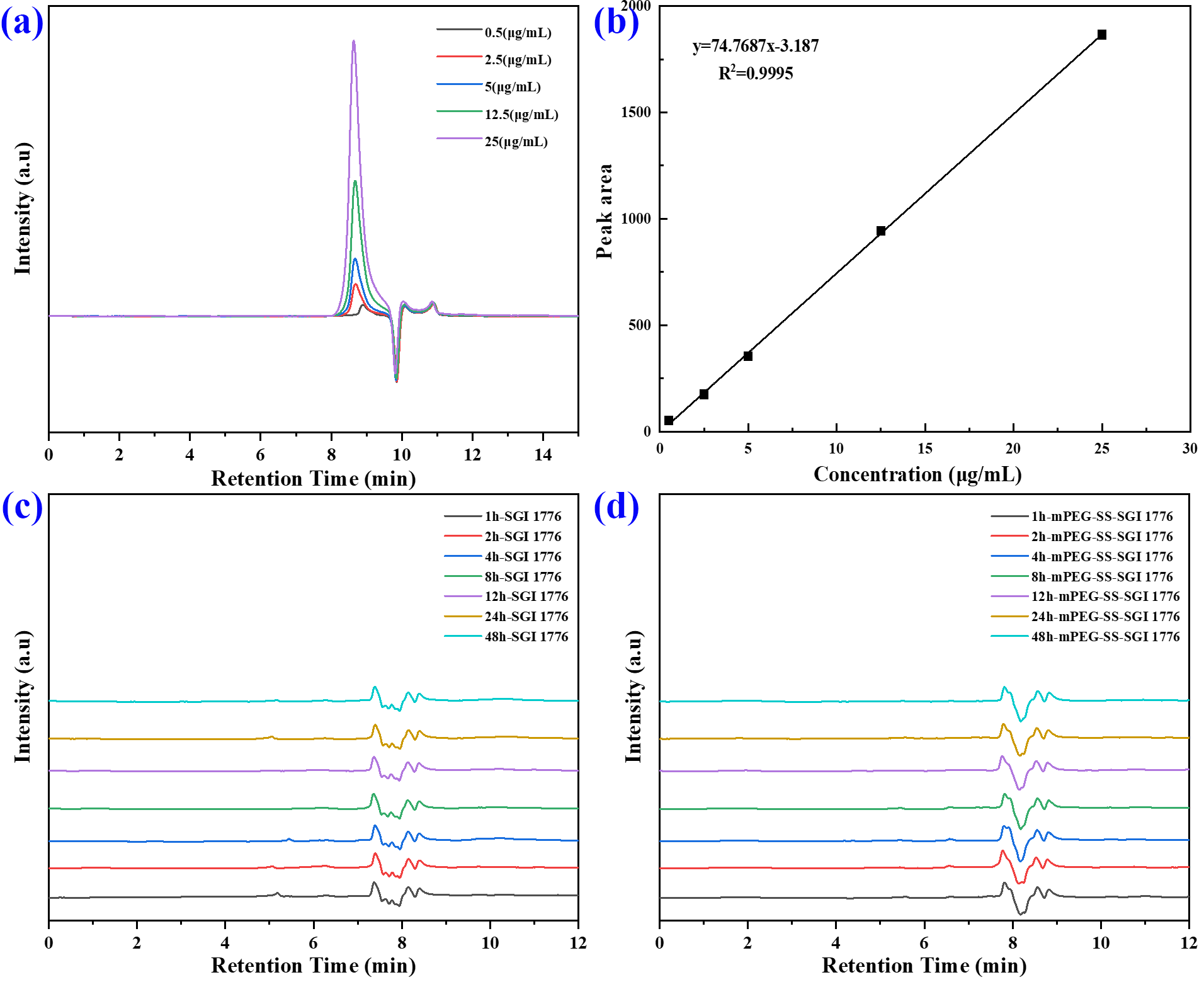


**Supplementary Figure 8.** (a) HPLC curves of different concentrations of SGI1776 standard solution in blank plasma. (b) Calibration curve generated using standard SGI1776 concentration measured using HPLC. HPLC profiles of free SGI1776 tablets (c) and conjugate (d) in plasma at different times.


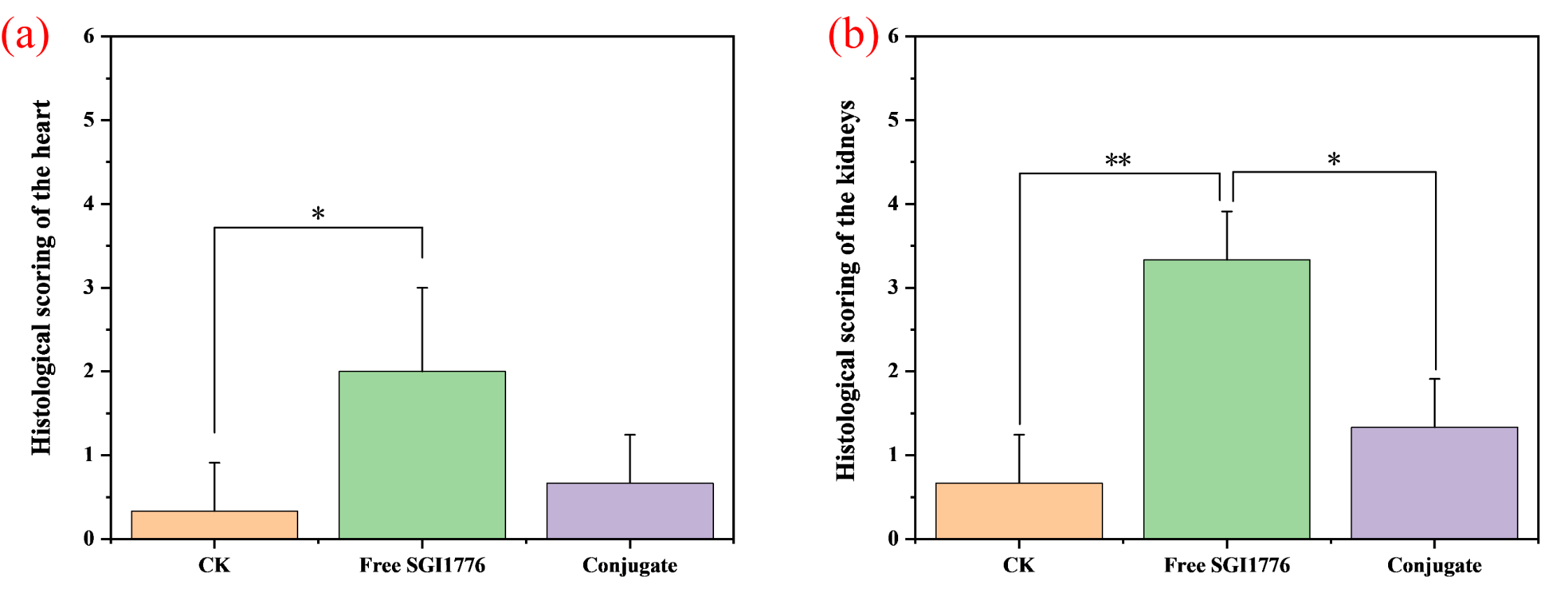


**Supplementary Figure 9.** Histological scoring of the heart (a) and kidneys (b). (*p < 0.05, **p < 0.01, ***p < 0.001)
